# SIRT3 Functions as an Eraser of Histone H3K9 Lactylation to Modulate Transcription for Inhibiting the Progression of Esophageal Cancer

**DOI:** 10.1016/j.mcpro.2025.100973

**Published:** 2025-04-17

**Authors:** Chen Chen, Yingao Zhang, Yong Zang, Zilong Fan, Yanpu Han, Xue Bai, Aiyuan Wang, Jianji Zhang, Ju Wang, Kai Zhang

**Affiliations:** 1 Key Laboratory of Breast Cancer Prevention and Therapy (Ministry of Education), Key Laboratory of Immune Microenvironment and Disease (Ministry of Education), The Province and Ministry Co-sponsored Collaborative Innovation Center for Medical Epigenetics, School of Basic Medical Sciences, Tianjin Medical University Cancer Institute and Hospital, Tianjin Medical University, Tianjin, China; 2 School of Biomedical Engineer, Tianjin Medical University, Tianjin, China; 3 Department of Bioinformatics, Tianjin Key Laboratory of Medical Epigenetics, Tianjin Medical University, Tianjin, China; 4 State Key Laboratory of Experimental Hematology, Tianjin Medical University, Tianjin, China

**Keywords:** proteomics, histone lactylation, epigenetics, gene transcription, esophageal squamous cell carcinoma

## Abstract

Lysine lactylation (Kla) links lactate metabolism to epigenetic regulation, playing a key role in modulation of gene expression in tumor and immune microenvironment. Our recent study shows that HBO1-mediated histone H3K9la activates the transcription of genes encoding tumorigenesis, suggesting the potential significance of intervening in this Kla site for tumor therapy. Evidence so far indicates that traditional deacetylases can catalyze the removal of Kla; however, the precise demodifying enzyme to histone H3K9la *in vivo* and functional consequence remain elusive. Herein, we combined an antibody-based proximity labeling approach with mass spectrometry analysis to identify SIRT3 as a major binder to histone H3K9la and showed the specific catalysis of SIRT3 for the removal of lactylation. Molecular docking further revealed the molecular mechanism of the binding of histone H3K9la to SIRT3. More importantly, SIRT3 can specifically modulate gene transcription by regulating H3K9la, inhibiting the progression of esophageal squamous cancer cells. Together, our work identifies the specific delactylase of H3K9la and reveals an H3K9la-mediated molecular mechanism catalyzed by SIRT3 for gene transcription regulation in esophageal squamous cancer cells, and our findings provide an opportunity to investigate the physiological significance of Kla controlled by SIRT3 in cancer.

## Highlights

• Identifying H3K9la interactors by an antibody-based proximity labeling approach.
• SIRT3 can erase specifically histone H3K9la to regulate gene transcription.
• H3K9la-mediated molecular mechanism for inhibiting the progression of ESCC.

## Graphical Abstract

**Figure:**
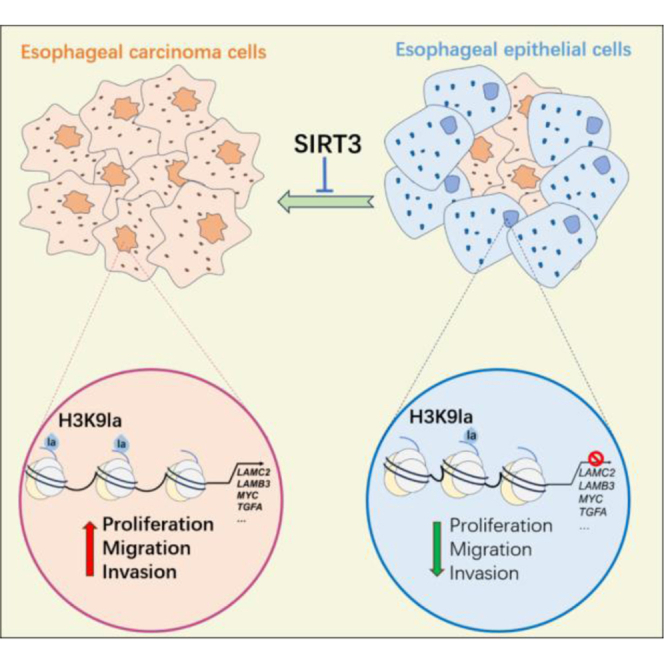

## In Brief

This study mapped the interactors of histone H3K9 lactylation (H3K9la), using an antibody-based proximity labeling approach and mass spectrometry analysis. SIRT3 was identified as a specific delactylase for H3K9la and molecular docking elucidated the structural basis of their interactions. Findings further show that SIRT3 can specifically modulate gene transcription by regulating H3K9la, inhibiting the progression of esophageal squamous cancer cells. This work not only reveals an H3K9la-mediated molecular mechanism catalyzed by SIRT3 for gene transcription regulation, but also proposes a novel therapeutic avenue targeting lactylation metabolism in tumors.

Chromatin structure and transcriptional activity of genes are regulated by histone posttranslational modifications (PTMs) (1, 2). Increasing evidence indicates that the status of histone marks is related to cell metabolism (3), and the metabolites (4) and their intermediates (5) can be used as signaling molecules to transmit intercellular or intracellular signals (6). Short-chain fatty acids can be produced by cellular metabolism or by fermentation from the gut microbiota (7). These metabolites can act as precursors of acyl-CoA for histone acylation (8). Lactate was considered a metabolic waste for a long time, but has recently been found to play an important role as an energy substance in metabolism (9, 10). Lactate is closely related to many cellular processes and the occurrence and development of a variety of diseases, such as cancer (11), diabetes (12), cirrhosis (13) and other diseases (14). Recently the nonmetabolic functions of lactate in physiology and diseases attract interest.

Zhang *et al* (15) reported in 2019 that lactate, as a precursor, can affect gene transcription and expression by loading onto the side chain of histone lysine residues through acyltransferases such as p300, HBO1 (histone acetyltransferases binding to ORC1) or YiaC (16, 17). In addition, lactylation modified on the ε-group of lysine is distinguished from acetylation by its branched, chiral, and four-carbon length properties (15, 18). In the process of glycolysis, the pathogenesis of histone Kla and Kac is also distinguished (19). Therefore, histone Kla is a physiologically relevant histone modification mark with unique biological functions. Various studies have shown that histone lactylation is involved in transcriptional regulation (20, 21, 22). However, the regulatory mechanisms of histone lactylation remain to be explored, including the removal of key lactylated sites in the cell. Understanding the roles of various classes of acyltransferases and deacylases in regulating lysine acylation will promote us to more fully understand their roles in cell regulation and chromatin signaling pathways. HDAC1-3 are the erasers with delactylase activity reported (23), and NAD^+^ dependent deacetylases sirtuins (SIRTs) share conserved central catalytic domains as possible regulators involved in different epigenetic programs (18, 24). Among them, SIRT2 (25, 26) and SIRT3 (27, 28, 29) have been reported to have potential delactylation enzyme activity for histones, in particular, SIRT2 can delete lactylation at histone H2AK115, H2BK85, H3K18 and H4K8, H4K12 and H4K91 while, SIRT3 shows a preference for delactylation at H4K16 and H3K23. Besides, SIRT3 is a nonhistone delactylase of CCNE2. SIRT1 and SIRT3 are delactylase of H4K5, H4K8, and H4K12. Delactylase effects of SIRT1 on a positive feedback loop involve the H19-glycolysis-histone lactylation in gastric cancer (30). These studies show that traditional deacetylases may remove lactylation, indicating the influence of these enzymes on lactylome; however, a specific Kla site may be targeted by several delactylases or none. Herein, the precise removal of histone H3K9la remains to be explored

The importance of histone H3K9 PTMs, such as methylation and acetylation, has been fully recognized. For examples, H3K9me can selectively block transcription factor activity to ensure differentiated tissue integrity (31). Kla on H3K9 has been found to regulate different kinds of diseases, H3K9la confers Temozolomide resistance in glioblastoma *via* LUC7L2-mediated intron 7 retention of MLH1 (32). With the high expression of glucose transporter 3 (GLUT3) glucose metabolism is accelerated, which results in the generation and accumulation of large amounts of lactate. Excess lactate acts as a precursor for Kla and increases H3K9la level to regulate the occurrence and progression of gastric cancer (33). We recently found that HBO1-mediated H3K9la may activate the transcription of genes encoding tumorigenesis and showed the potential effect of histone H3K9la on the malignant behaviors of multiple cancer cells, including esophageal squamous cancer cells (ESCC) (17). Esophageal cancer (EC) is a major life-threatening health problem around the world. ESCC accounts for 90% of ECs and is highly prevalent in the East. There is an obvious need for systemic treatment to improve the progression of ESCC, focusing on molecular targeted therapy (34, 35). It is also important to investigate genes associated with development of ESCC and characterize their roles in the targeted therapy. However, the regulatory elements and functional consequence of H3K9la in ESCC are both unclear. To date, it is still a challenge to search for regulatory factors of specific histone mark *in vivo* due to technical limitations. An antibody-mediated proximity labeling strategy with fusion proteins Protein A-TurboID, which does not require molecular cloning or transfection of target cells, has been developed for profiling of *in vivo* interactors of the target (36).

Here, we systematically identified the histone H3K9la interactors including potential delactylation enzymes, by using an antibody-mediated protein A-TurboID proximity labeling method. We further found that SIRT3 is an active lysine delactylation enzyme *in vitro* and also has intracellular delactylation activity. In addition, knocking out SIRT3 or using the specific inhibitor 3-TYP can directly affect intracellular delactylase activity or histone Kla levels in ESCC cells. Our data suggest that SIRT3 is a bona fide H3K9la delactylase in cells. Therefore, our study of SIRT3 histone delactylation suggests a potential regulatory mechanism between lactate kinetics and gene regulation under metabolic changes, acting in multiple biological pathways in response to nutrient and environmental perturbations and maybe the target for the EC therapy.

## Experimental Procedures

### Cell Lines, Antibodies, and Reagents

Human EC cell line KYSE30 and human embryonic kidney cell line HEK 293T were cultured in Dulbecco’s modified Eagle’s medium (VivaCell). All cells were cultured in medium supplemented with 10% fetal bovine serum, 100 U/ml penicillin, and 100 mg/ml streptomycin in 5% CO_2_ at 37 °C. The following antibodies were used in this study: Anti-L-Lactyl-Histone H3 (Lys9) Rabbit mAb (PTM-Biolabs 1419RM), SIRT3 Polyclonal antibody (Proteintech 10099-1-AP), Histone H3 Rabbit pAb (Abclonal A2348), VDAC1 antibody (Proteintech 55259-1-AP), Mouse anti DDDDK-Tag (Flag) monoclonal (Abclonal AE005), Mouse anti-alpha tubulin monoclonal antibody (Yeasen 30304ES60). Hifair AdvanceFast One-step RT-gDNA Digestion SuperMix for qPCR (Yeasen 11151ES10), Hieff qPCR SYBR Green Master Mix (No Rox) (Yeasen 11201ES03). Cell Counting Kit-8 (New Cell & Molecular Biotech, C6005).

### Recombinant Protein A-TurboID Fusion Protein Expression and Purification

Protein A-TurboID fusion gene was chemical synthesized and inserted in the pET-22b vector. A His tag was contained to the C-terminal part of the protein for affinity purification. The pET-22b-Protein A-TurboID plasmid was transformed into *Escherichia coli* BL21 (DE3). Monoclonal colonies were grown overnight and cultured in 2L LB medium until reaching *A*_600_ 0.6. Protein expression was then induced with 0.5 mM IPTG for 16 h. The bacteria were harvested, resuspended in 100 ml of lysis buffer (50 mM Tris–HCl pH 8.0, 500 mM NaCl, 5% glycerol, and 1 mM PMSF), and sonicated. The lysate was centrifuged for 30 min at 16,000*g* at 4 °C to harvest supernatant protein. The protein extract was then passed through a Ni-NTA (Cytiva) chromatography column (10 ml, Bio-Rad) by gravity flow, and the column was then washed three to five times with washing buffer (50 mM Tris–HCl pH 8.0, 500 mM NaCl, 5% glycerol, and 20 mM imidazole). The Protein A-TurboID fusion protein was then eluted with 15 ml of elution buffer (50 mM Tris–HCl pH 8.0, 500 mM NaCl, 5% glycerol, and 300 mM imidazole), then stored in buffer (20 mM Tris–HCl pH 8.0, 150 mM NaCl) at −80 °C. Aliquots of the collected protein were loaded on an SDS–PAGE and stained to determine the amount and purity.

### Streptavidin Affinity Enrichment of Biotinylated Proteins

Cells were cultured in 15 cm dishes and washed with PBS once and then fixed in 3 ml PBS containing 4% paraformaldehyde for 15 min at room temperature (RT). Cells were then collected and centrifuged at 150*g* for 3 min at RT. Cell pellets were resuspended and incubated in 1 ml of lysis buffer (10 mM Tris–HCl pH 7.5, 10 mM NaCl, 3 mM MgCl_2_, 0.3% NP40, and 10% glycerol) for 10 min on ice, and then centrifuged at 800*g* for 8 min at 4 °C to isolate the nuclei. Nuclei were then washed three times with 0.5 ml of lysis buffer and centrifuged at 200*g* for 2 min at 4 °C. Nuclei were then washed once with 0.5 ml of PBS and resuspended to permeabilize in 1 ml of PBS supplemented with 0.3% Triton-X100 and rotated for 10 min. Nonspecific-binding sites were blocked with 1 ml PBS containing 0.3% Triton-X100 supplemented with 3% bovine serum albumin for 30 min and rotated at RT. Samples were then incubated with 2 μg of primary antibody (immunoglobulin G (IgG) rabbit antibody and H3K9la antibody) diluted in 300 μl of blocking buffer and rotated for 1 h at RT. Nuclei were washed twice with 500 μl of PBS to remove the unbound antibody and were incubated with 3.4 μg of Protein A-TurboID protein diluted in 300 μl of blocking buffer for 1 h and rotated at RT. The pellet was washed twice with 500 μl of PBS to remove the unbound Protein A-TurboID protein. Next, samples were incubated with 300 μl of biotin reaction buffer (5 mM MgCl_2_, 5 μM biotin, and 1 mM ATP in PBS) and in 1000 rpm shaker for 10 min at 37  °C. Samples were then centrifuged, washed once with 1 ml PBS and finally lysed in 300 μl of radioimmunoprecipitation assay (RIPA) buffer (50 mM Tris–HCl pH 7.8, 150 mM NaCl, 0.5% NaDC, 0.1% SDS, and 1% NP40) overnight on ice. The following day, samples were sonicated using a sonicator (10 cycles of 1s on and 1s off) and then decrosslinked for 1 h at 95 °C by adding 1% SDS. Samples were centrifuged at 21,000*g* for 10 min at 4 °C. Supernatant was then incubated with 12.5 μl high-performance streptavidin sepharose beads (15511301, Thermo Fisher Scientific) and rotated for 2 h at 4 °C. Beads were then washed once with 0.5 ml RIPA buffer and three times with 500 μl PBS. One microliter of fresh trypsin was then added to each sample and incubated overnight at 37 °C. The following day, peptides were acidified by adding 10 μl of 10% TFA and purified on C18 Ziptips (3M Empore), and further analyzed by LC–MS/MS (Eclipse, Thermo Fisher Scientific).

### LC-MS/MS Analysis

After desalting, each tryptic digest was redissolved in HPLC solvent A (0.1% (v/v) formic acid in water) and injected into a nano-LC system (EASY-nLC 1200, Thermo Fisher Scientific). Each sample was separated by a C18 column (75 μm inner-diameter × 15 cm, 3 μm) at a flow rate of 300 nl/min. The HPLC gradient was as follows: 5% to 10% solvent B (0.1% formic acid in 100% acetonitrile) in 15 min, 10% to 22% solvent B in 45 min, 22% to 35% solvent B in 15 min, 35% to 90% solvent B in 5 min and hold for 10 min at 90% solvent B. The HPLC elute was electrosprayed directly into an Orbitrap Q Exactive mass spectrometer (Thermo Fisher Scientific). Spray voltage was set to 2.4 kV and ion transfer tube temperature 320 °C. The orbitrap mass analyzer was used as the MS1 detector with 70,000 resolution and scan range 350 to 1750 m/z. The automatic gain control target was set to 3e6 for MS1, and to 5e4 for MS2; the maximum injection time was set to 50 ms in MS1 and to 100 ms in MS2. The orbitrap mass analyzer was used as the MS2 detector with 17,500 resolution and scan range 200 to 2000 m/z. Precursor ions with charges of +2 to +5 were isolated for MS2, and dynamic exclusion time was set at 20 s. The MS2 isolation window was 2.0 m/z, loop count was 15 and NCE was set 27% for precursor fragmentation.

### Data Analysis

The resulting MS/MS data were searched using Thermo Proteome Discoverer (ver. 3.0), the Swiss-Prot human proteome database in FASTA format excluding protein isoforms (downloaded on 12.18.2022, 20,389 sequences) was used with the overall false discovery rate for peptides of less than 1%. Trypsin specificity was applied for peptide sequences search allowing a maximum of two missed cleavages. Carbamidomethylation on cysteine was set as fixed modification. Methionine oxidation and acetylation on protein N terminal were set as variable modifications. Mass tolerances for precursor ions were set at ±10 ppm for precursor ions and ±0.02 Da for MS/MS fragments. A label-free protein quantification was performed. At least two peptides were required for a protein identified from data. Peptides were quantified using the MS1 intensity, and peptide abundance values were summed to yield the protein abundance values. In Proteome Discoverer (ver. 3.0), normalization mode parameter was set to total peptide amount, and the scaling mode parameter was set to On All Average. Annotated proteins identified in this study can be found in supplemental data S1. The mass spectrometry (MS) proteomics data have been deposited to the ProteomeXchange Consortium (https://proteomecentral.proteomexchange.org) *via* the iProX partner repository (37, 38) with the dataset identifier PXD061251.

### Molecular Docking

The structure of peptide H3K9la (7–12aa) was retrieved using the software Chem3D for further molecular docking. Protein Data Bank was used to download the receptor protein. Here, SIRT3 (Protein Data Bank: 3GLS) was taken as the target receptor proteins. The proteins were subjected to the AutoDock Tool 4.2 (39, 40). To perform the steps of protein preparation. AutoDock Vina Software was used to perform molecular docking of ligand with proteins. AutoDock Vina comes with the feature of the calculation of grid maps automatically. After the successful docking, confirmation of the binding position of ligand into the receptor proteins and calculation of bond distance has been done by PyMOL (www.pymol.org) and LIGPLOT. PyMOL and LIGPLOT allow the clear visualization of binding of ligand-protein with its hydrogen bonds as well as bond distance and hydrophobic interactions.

### Recombinant hSIRT3 Protein and Histone Peptide Preparation

Full-length hSIRT3 gene was optimized and synthesized by Company (GentleGen), the genes (wild-type (WT) and mutant H248Y, V324M, and D346A) were cloned into overexpression vector pLVX-CMV in mammalian HEK 293T cells for transient expression (Life-iLab, AC04L091).

Truncated hSIRT3 (NAD^+^ dependent deacetylase domain, 118–399 aa) was cloned into a pET-28b-SUMO vector (modified based on pET28b) containing N-terminal 6xHis-SUMO tag. WT SIRT3 and mutants were overexpressed in *E. coli* BL21 (DE3) strain. Cells were cultured in LB medium supplemented with 0.1 mM ZnCl_2_ and induced with 0.5 mM isopropyl β-d-thiogalactoside (IPTG) at 16 °C overnight. The cells were resuspended in buffer A (20 mM Tris–HCl pH 7.5, 500 mM NaCl, and 5% glycerol) and then lysed with a sonicator. The supernatant was further obtained by centrifugation and loaded into a nickel column preequilibrated with lysis buffer. After the buffer A containing 30 mM imidazole was washed, the His-SUMO tag was cut overnight on the column by ULP protease. SIRT3 protein was eluated, collected, ultrafiltration and stored at −80 °C for subsequent use. All mutant SIRT3 proteins were purified using essentially the same procedure. All SIRT3 mutants were generated by QuikChange site-directed mutation strategy (Genstar) and verified by sequencing.

The histone peptides used in this study contain the first 17 amino acids from H3 with or without lactylation and acetylation on lysine 9 (H3K9la and H3K9ac) followed by two glycines and a biotinylated lysine were synthesized by Scilight-Peptide company and the purity was more than 95%. The purity and identity of the peptides were verified by LC-MS.

### Isothermal Titration Calorimetry

The isothermal titration calorimetry (ITC) experiments were carried out on the MicroCal PEAQ ITC instrument (Malvern Instrument) at 25 °C. The 100 μM protein was dropped into the 1000 μM peptide segment for 19 consecutive drops, and the resulting titration curve was drawn using the "one set of binding sites" model and the Origin 7.0 program. The protein concentration was determined by UV absorption at 280 nm. The peptide concentration was measured by nanodrop.

### Peptide Pulldown

Peptides were dissolved in Buffer A (20 mM Tris–HCl pH 8.0, 150 mM NaCl, 0.1% NP40) and incubated with 75 μl of high-performance streptavidin sepharose beads (15511301, Thermo Fisher Scientific) previously equilibrated with Buffer A. Beads were then washed three times with Buffer A and incubated with 10 μg SIRT3 (WT and mutants) in a rotation wheel at 4 °C overnight. After incubation, beads were washed three times and collected, 20 μl SDS loading buffer was added and incubated for 5 min at 95 °C and loaded on an SDS–PAGE gel for Western blot.

### *In vitro* Delactylation Assays

Enzymatic activity of hSIRT3 was measured by detecting the removal of lactyl groups from the peptide. The peptides were analyzed by liquid chromatography-tandem mass spectrometry (LC-MS/MS), MALDI-TOF MS and dot blot experiment, respectively. For LC-MS/MS analysis, SIRT3 protein (5 μM) was incubated with the corresponding 500 μM H3K9la peptide and 1 mM NAD^+^ in a reaction buffer containing 20 mM Tris–HCl pH 7.5 and 1 mM DTT at 37 °C for 2 h. Equal volume of 20% (v/v) TFA was added to stop the reaction. Samples without NAD^+^ or enzymes were treated under the same conditions as controls. The samples were spun for 10 min at 18,000g to separate the enzymes from the reaction. The remaining samples were separated by a C18 column (75 μm inner diameter × 15 cm, 3 μm beads) at a flow rate of 300 nl/min in HPLC gradient: 5 to 50% buffer B (0.1% formic acid in acetonitrile) in 10 min, 50 to 100% buffer B in 17 min and keeping at 100% for 3 min and then analyzed by Orbitrap Q Exactive mass spectrometer (Thermo Fisher Scientific). From the analysis of LC-MS/MS, we extracted ion chromatograms of the peptides and observed that the signal intensities of H3K9la reduced.

For the delactylation activity study by MALDI-TOF MS, the reaction system combined 1 μM SIRT3, 100 μM H3K9la peptide, and 5 mM NAD^+^ in a buffer containing 20 mM Tris–HCl pH 7.5 and 1 mM DTT. After incubation at 37 °C, the reaction was terminated by adding the final concentration of 5% TFA, and the reaction system without enzyme was used as control. The reaction mixture was then desalted by ZipTip and analyzed by MALDI-TOF MS (Atouflex Speed). In the analysis of MALDI-TOF MS, we observed an obvious mass shift of 72.021 Da on H3K9la peptide.

For the dot blot experiment, 500 ng histone peptide H3K9la was first added to the nitrocellulosic membrane, and then air dried at 37 °C. Concentration gradient of SIRT3 (0, 0.01, 0.1, 1, and 10 μM) in the reaction buffer (20 mM Tris–HCl pH 7.5, 100 mM NaCl, 1 mM DTT, and 5 mM NAD^+^) were added to the peptide dot. After reaction, anti-H3K9la antibodies (PTM Biolabs) were used to monitor residual H3K9la level on the membrane.

### Histone Extraction and Delactylation Activity

Histone was isolated from HEK 293T cells by acid extraction. In short, HEK 293T cells were treated with 20 mM sodium lactate for 24 h and harvested by centrifugation. The harvested HEK 293T cells were resuspended with lysis buffers (10 mM Tris–HCl pH 8.0, 1 mM KCl, 1.5 mM MgCl_2_, 1 mM DTT, 2 mM PMSF, and Roche Complete EDTA free protease inhibitor) and incubated at 4 °C for 1 h. The intact nuclei were centrifuged at 2000*g* at 4 °C for 10 min to form pellets. To extract histones, 0.4 M H_2_SO_4_ was added to the suspension nucleus and then rotated overnight at 4 °C. After removal of nuclear debris by centrifugation at 10,000*g* at 4 °C for 10 min, histone was precipitated by adding 100% trichloroacetic acid (trichloroacetic acid final concentration 33%). The precipitated histones were incubated at 4 °C for 10 min and washed with acetone twice. The dried protein particles were dissolved with ddH_2_O. Subsequently, 4 ∼ 6 μg histone was incubated with 10 μM SIRT3 in 100 μl PBS supplemented by 5 mM NAD^+^ at 37 °C for 5 h. The lactylation levels at histone were detected by Western blot.

### Nucleosome Extraction and Delactylation Activity

To detect the delactylation activity of SIRT3 to nucleosomes. Lactylation nucleosomes were generated in HEK 293T cells by adding 20 mM sodium lactate for 24 h and harvested by centrifugation. The cells were resuspended in cold extraction buffer (15 mM Tris–HCl pH 7.5, 15 mM NaCl, 60 mM KCl, 2 mM EDTA, 0.5 mM EGTA, 0.5 mM spermine, 0.15 mM spermidine, 0.34 M sucrose, 15 mM β-mercaptoethanol, 0.5% Triton X-100, and 0.2 mM PMSF) at 4 °C for 10 min. The lysate was centrifuged at 2500*g* at 4 °C for 10 min to remove the cytosolic components. The chromatin pellets were washed three times with extraction buffer and resuspended in chromatin MNase digestion buffer (0.2 units/μl MNase, 50 mM Tris–HCl pH 7.5, 25 mM KCl, 4 mM MgCl_2_, and 1 mM CaCl_2_). The mixture was centrifuged at 4000*g* at 4 °C for 10 min. The pellets were collected and resolved in a dissolving buffer (1 mM Tris–HCl pH 7.8, 0.2 mM EDTA, and 0.1 mM PMSF) on ice for 1 h. The solution was centrifuged at 4000*g* at 4 °C for 10 min, and the supernatant containing nucleosomes was collected for delactylation reaction. Briefly, 7 μM nucleosomes were incubated with 10 μM SIRT3 in 20 μl reaction buffer (20 mM Tris–HCl pH 7.8) supplemented by 5 mM NAD^+^ at 37 °C for 5 h. The lactylation levels in nucleosome were detected by Western blot.

### Extraction of DNA from Nucleosome

Subsequently, 10 μl extraction buffer (phenol: chloroform: isoamyl alcohol = 25: 24: 1, pH 7.8) was added to 50 μl nucleosome solution. The upper layer solution was collected. The extraction process was repeated once. Precipitation buffer (3 M sodium acetate (NaAc), pH 5.2 and cold alcohol) was added to the collected buffer to precipitate DNA at −20 °C for 20 h. The mixture was centrifuged at 12, 000*g* at 4 °C for 30 min. The pellet was collected and air dried for 5 min. The DNA pellet was dissolved in double distilled water and centrifuged at 12, 000*g* at 4 °C for 30 min. The DNA solution was analyzed by 2% agarose gel.

### Nuclear and Cytosolic Fractionation Analysis

The KYSE30 cells were centrifuged at 500*g* for 2 min at RT. The cell pellet was used for cytoplasmic separation. The cell pellet was washed with 300 μl cold PBS containing 0.4% NP40 for 5 min on ice and centrifuged at 5000 rpm for 10 min at 4 °C, the preserved supernatant was cytoplasmic proteins. Particles in the wash buffer pass through the 1000 μl pipette head 5 times to effectively remove perinuclear mitochondria from the nucleus. The dissolved pellets were centrifuged again at 15,000*g* for 10 s and the supernatant was discarded. Finally, 75 to 100 μl PBS containing 0.1% NP40 was added into the nuclear sphere, and the pipetting was repeated with the tip of a 200 μl needle to serve as the nuclear fraction. VDAC1 antibody was used as control to test for mitochondrial contamination in the nuclear fraction. SIRT3 KO KEYSE30 cells were treated as negative control.

### Colocalization Immunofluorescence Analysis

KYSE30 cells were fixed with 4% (w/v) paraformaldehyde PBS for 10 min, infiltrated with 0.2% Triton X-100 at RT for 5 min, and washed with PBS. SIRT3 antibody was incubated at 4 °C overnight and secondary antibody was incubated for 1 h at RT. The nuclear fractions were stained with 4',6-diamidino-2-phenylindole. Fluorescence images were taken with the confocal laser scanning microscope system. The projected images are generated by the ImageJ software. SIRT3 KO KEYSE30 cells were treated as negative control.

### CRISPR-Cas9 Establishment of SIRT3 KO KYSE30 Cells

The target single-guide RNA was cloned into the lenti-CRISPR v2 vector and cotransfected with psPAX2 and pMD2G plasmids to generate the corresponding virus in HEK 293T cells. After infecting the target cells required by the virus for 36 h, the cells were screened with 2 μg/ml of purinomycin until no cells died. The primers for SIRT3-KO are listed below.

SIRT3-sg-forward: GTTCTGGGGTTGGCGCGCCG

SIRT3-sg-reverse: CGGCGCGCCAACCCCAGAAC

### ChIP-Seq and ChIP-qPCR

Briefly, 2 × 10^7^ cells were fixed with 1% formaldehyde at RT for 10 min, and the fixation was stopped by 125 mM glycine at RT for 5 min. The chromatin pellets were extracted from cells by lysis buffer (10 mM Tris–HCl pH 7.4, 10 mM NaCl, 3 mM MgCl_2_, and 0.5% NP-40), and resuspended with 0.8 ml of RIPA buffer. The genomic DNA was fragmented by sonication at 25% amplitude for a total of 8 min with intervals. Sonicated chromatin was incubated with 2 μg of anti-H3K9la antibody, or control IgG precoated on Dynabeads protein G at 4 °C overnight. For ChIP-seq data processing, the ChIP-seq reads were aligned to the hg19 reference genome using Burrows-Wheeler Alignment tool v0.7.12. After filtering duplicates, multimappers, ENCODE blacklist regions, MACS2 2.7.1 was used to call the peaks with the following parameters. Results from the ChIP-seq data were visualized in Integrative Genomics Viewer v2.8.1, and the peaks were annotated by PAVIS 14. The promoter region was defined as ± 3kb around the known transcriptional start site.

For chromatin precipitation quantitative PCR (ChIP-qPCR), after washing the immunoprecipitants with RIPA buffer and LiCl washing buffer (40 mM Tris–HCl pH7.9, 1 mM EDTA, 250 mM LiCl, 0.5% NP-40, 0.5% Na-deoxycholate), the chromatin was crosslink reversed by protease K digestion at 65 °C overnight and purified using a quick PCR purification kit (Genstar). The immunoprecipitated DNA fragments were then assessed by qPCR as described above with the primer information being listed below.

*MYC* Forward: TTTCCTCCACTCTCCCTGGG

*MYC* Reverse: TAGCAGTACTGTTTGACAAACCGC

*LAMC2* Forward: TCTTAAGATTGGGCCTCCCAG

*LAMC2* Reverse: AGACAAACACACAGAGCACAAACC

*AREG* Forward: TTGACGTCATGGGCTGCG

*AREG* Reverse: AAAACGAGCGGGTGTTGGA

*EREG* Forward: TTCCTGACGGTCCTCCTGC

*EREG* Reverse: AGAGCGGCTGTCAGCCTAGA

*EPHA2* Forward: TTCCCGAAGTCGCCGCCT

*EPHA2* Reverse: AACCTACCTGGTGTCGCCCA

*TGFA* Forward: AAACTGGCAACACTCGGTCTAGG

*TGFA* Reverse: TCTAAGCCCCAGCGACGAG

*EFNA5* Forward: AACCGGATGCCAGCCAGA

*EFNA5* Reverse: ACCAACCGCTGGCGGCGC

*LAMB3* Forward: AATGATGGGCTGGGGCTC

*LAMB3* Reverse: AAAAGAGTTTGTGGCTGTCATTGG

### Wound Healing Assay

The wound healing assay involved seeding cells in 6-well plates. After the cells (KYSE30 cells with SIRT3 KO and overexpression) were fused, they were scraped off with the tip of the pipette, and the floating cells were washed away with PBS. Immediately afterward, the wounds were photographed. After culture in serum-free medium for 24 h, the wounds were photographed again.

### Transwell Migration and Invasion Assay

To perform the transwell migration and invasion assay, matrigel matrix was seeded in the upper chamber, and 40,000 cells (KYSE30 cells with SIRT3 KO and overexpression) were mixed in 200 μl serum-free medium and seeded in the upper chamber. Then 600 μl of complete medium was added to the bottom chambers. After incubation for 24 h, the cells were fixed for 30 min, and then stained with crystal violet for 1 h. After cleaning and drying, the cells were photographed under a microscope and counted using the ImageJ software.

### Cell Clonal Formation Assays

The cells (KYSE30 cells with SIRT3 KO and overexpression) to be observed were counted, and 1000 cells/well were inoculated into the 12-well plate culture plate, and the medium was changed every 3 days. About a week after cell culture, the cells were washed twice with PBS and fixed with 1 ml 4% paraformaldehyde for 10 min. The cells were stained with crystal violet solution for 5 to 10 min, and the excess crystal violet solution was washed away with PBS. The formation of cell clones was photographed. The results were analyzed by ImageJ software (v.ImageJ2).

The cells were counted and 1000 cells were inoculated in 6-well plate plates, and the medium was changed every 3 days. After about 1 week of cell culture, the cells were washed twice with PBS and fixed with 1 ml 4% paraformaldehyde for 30 min. Dye crystal violet for 5 to 10 min and then the cells were washed with PBS. The formation of cell clones was photographed and analyzed using ImageJ software.

### ESCC Xenograft Animal Model Assays

BALB/c female nude mice aged 4 to 6 weeks were purchased and fed adaptive for 1 week. Almost 2 × 107 KYSE30 cells (SIRT3 KO cells and Vector cells) were injected into nude mice. Tumor size was assessed every 4 days for 4 weeks. Tumor volume (mm^3^) = (length (mm)) × (width (mm))^2^ × 0.5. The tumors were dissected and weighed approximately 4 to 6 weeks after injection. The experimental animal program is authorized by the Scientific Research Animal Care and Use Committee of Tianjin Medical University. During the experiment, the researchers were unaware of the grouping of the mice. The sample size is described in the corresponding legend. No animals were excluded from the analysis.

### Experimental Design and Statistical Rationale

We employed an antibody-mediated Protein A-TurboID proximity labeling strategy to profile *in vivo* interactors of histone H3K9la, enabling rapid biotinylation of proximal proteins in the fixed cells. The workflow involved sequential incubation with H3K9la primary antibody (or IgG control) and Protein A-TurboID, biotin-triggered labeling, streptavidin bead enrichment, and quantitative MS to identify H3K9la-specific interactors. Triplicate technical replicates were analyzed to ensure robust identification of differential proteins associated with H3K9la. The total protein concentration of lysates was measured and equalized for blotting. For gene ontology (GO) term enrichment analysis, terms with Benjamini (adjusted *p*-value) values less than 0.001 are considered as enriched. Kyoto Encyclopedia of Genes and Genomes (KEGG) pathways with false discovery rate <0.05 is used for generating dot plot figures of ChIP-seq results. A *t* test was used in the study, and *p*-value smaller than 0.05 was considered as significantly regulated. All experiments performed in this manuscript were conducted as replicates.

### Statistical Analysis

Statistical analysis was performed with GraphPad Prism 8.0 software to assess differences between experimental groups. Statistical significance was analyzed by two-tailed *Student's t* test and expressed as a *p-value*. The difference between the groups was considered to be statistically significant when *p* < 0.05. ∗, ∗∗, and ∗∗∗ indicate *p* < 0.05, *p* < 0.01, and *p <* 0.001, respectively. ns indicates no significance.

## Results

### Profiling of Interactors of Histone H3K9la by an Antibody-Mediated Proximity Labeling Strategy

**Fig. 1:**
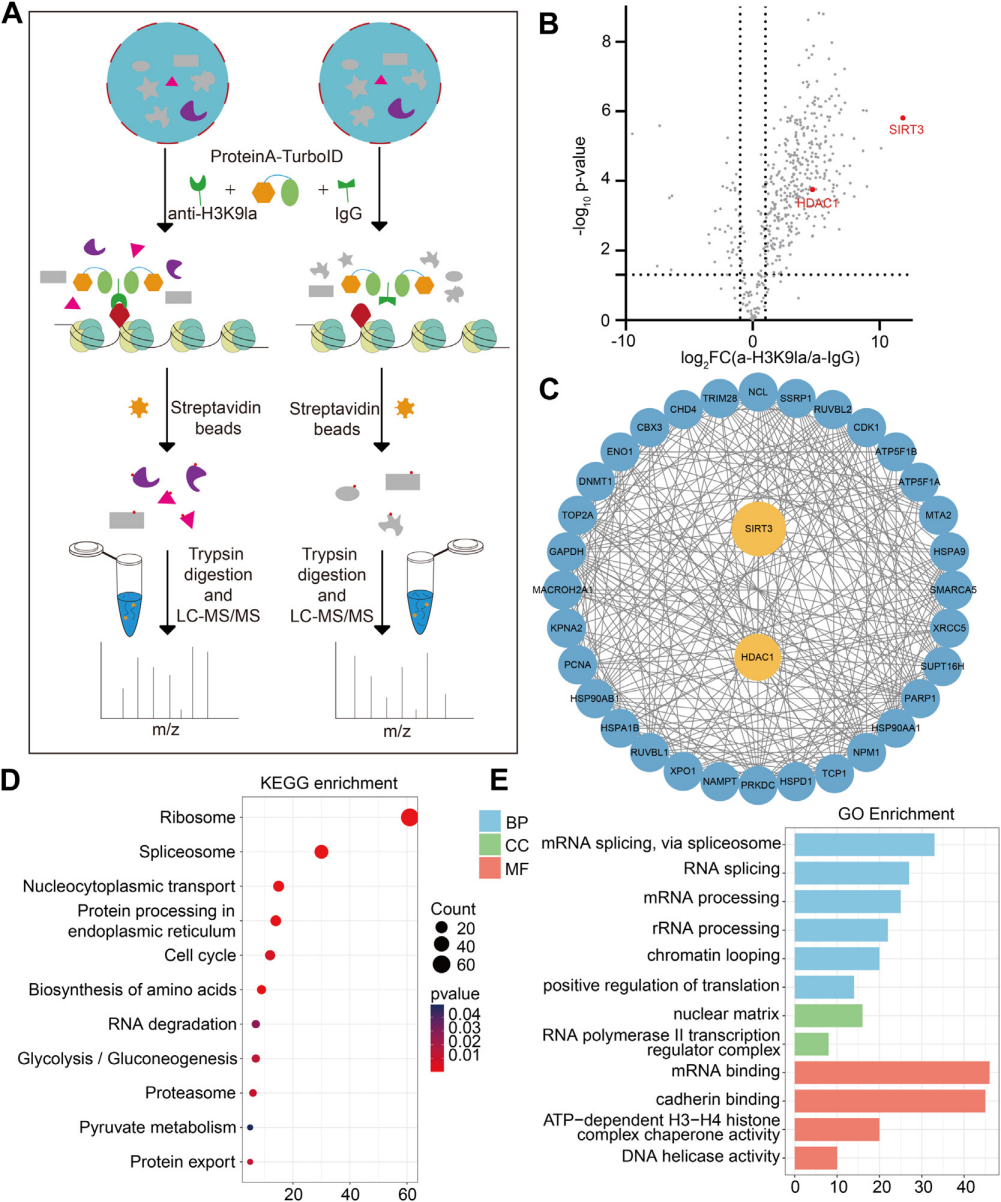
**Antibody-mediated protein A-TurboID proximity labeling strategy**. *A*, schematic diagram illustrating the antibody-mediated protein A-TurboID proximity labeling strategy to profile proteins that bind H3K9la with high affinity and selectivity in whole-cell proteomes. LC-MS, liquid chromatography–mass spectrometry. The *red dots* are corresponding to biotin. *B*, volcano plot of mass spectrometry analyses of biotin IPs. The proteins known to binding to the H3K9la are highlighted with *red dots*. The *x*-axis represent the log2 fold change (FC) and the *y*-axis the -log10 *p*-value. *C*, the association network of the proteins potentially interact with H3K9la by STRING analysis. *D*, GO analysis of the H3K9la potential binding proteins. *E*, KEGG pathways analysis of the H3K9la potential binding proteins. GO, gene ontology; KEGG, Kyoto Encyclopedia of Genes and Genomes.

We first sought to profile *in vivo* interactors of histone H3K9la by an antibody-mediated protein A-TurboID proximity labeling approach that enables proximity intracellular proteins biotinylation within minutes (36). We used an optimized workflow for profiling the interactors of target by means of Protein A-TurboID enzyme in fixed cells (Fig. 1*A*). First, the Protein A-TurboID fusion protein is expressed and purified from *Escherichia coli* as a fusion protein (supplemental Fig. S1*A*) that, when added to mammalian cell extracts, triggers protein biotinylation (supplemental Fig. S1*C*). After fixation with formaldehyde, the cells were infiltrated, and then histone H3K9la primary antibody and Protein A-TurboID enzymes were added. Meanwhile, using IgG antibody as a negative control, exogenous biotin was added to trigger the proximal protein biotinylation of the bait. Next, these biotinylated proteins are enriched by streptavidin beads and followed by proteomics analysis based on MS, respectively. Finally, the differential proteins were analyzed to determine the interactors preferred to H3K9la (Fig. 1*B*). In this experiment, three biological repeats quantitative mass spectrometry were performed, and a cluster of proteins were further identified to affinity enrich to H3K9la, including many positive control interactors, such as the reported delactylation enzymes HDAC1 and SIRT3 (Fig. 1*C* and supplemental Fig. S1, *B* and *D*). The GO and KEGG pathway enrichment analysis of affinity purification also showed the protein localization to nucleus and histone deacetylase complex items (Fig. 1, *D* and *E*). Among the preferred proteins, the nicotinamide adenine dinucleotide (NAD^+^)-dependent deacetylase SIRT3 was more than 10-fold enriched by H3K9la antibodies (supplemental Fig. S1*E*), indicating that it preferentially binds to this histone Kla. This result suggests that SIRT3 may be a selective and relatively tightly binding protein for histone H3K9la in nucleus.

### SIRT3 is Low-Expressed and Plays the Role of a Tumor Suppressor Gene in ESCC

**Fig. 2:**
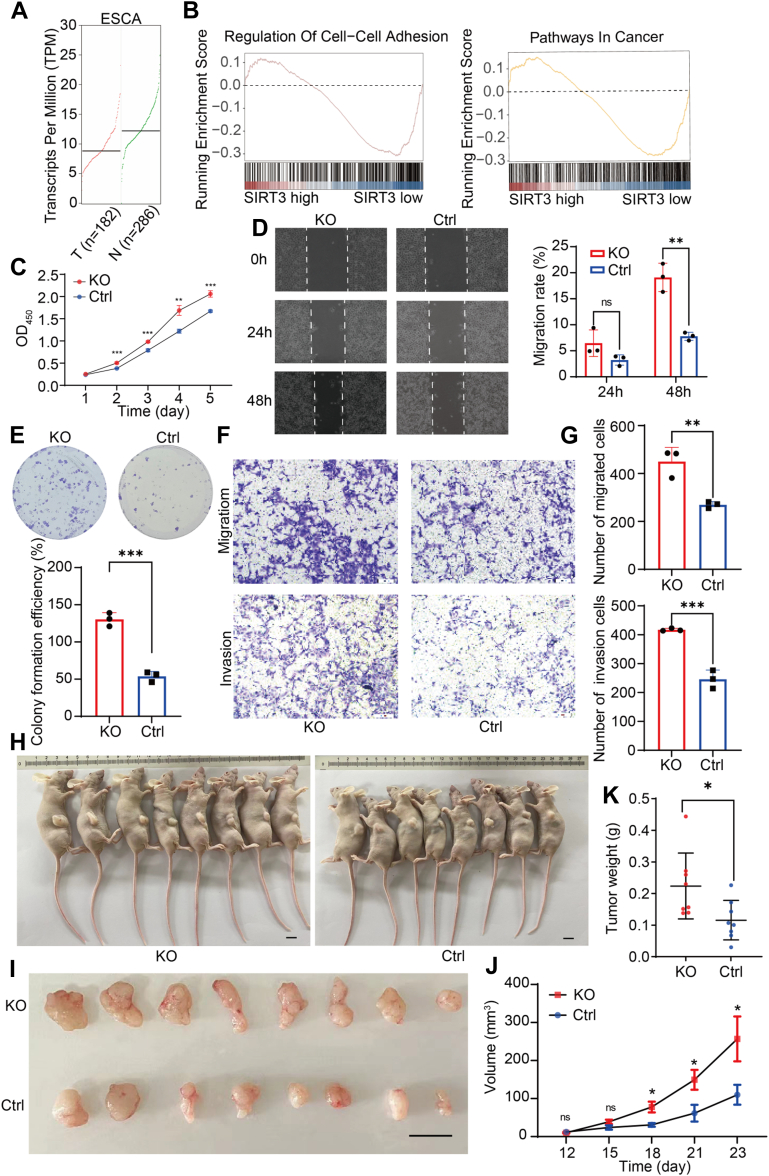
**SIRT3 is low-expressed and plays the role of a tumor suppressor gene in ESCC.***A*, SIRT3 expression is lower in ESCC tumor tissues than that in normal tissues analyzed in GEPIA. *B*, Gene Set Enrichment Analysis (GSEA) shows a significant expression change of genes for “regulation of cell-cell adhesion” and “pathways in cancer” (the associated genes in SIRT3 low expression ESCC cells were obtained from TCGA data). *C*, CCK-8 analysis of the viability of KYSE30 cells when knocking out SIRT3. The quantification of the A_450_ were shown in mean ± SEM (n = 8); ∗*p* < 0.05. Data are representative of three biological replicates. *D*, scratch wound healing assay results suggesting the impact of SIRT3 KO on the wound healing ability in KYSE30 cells (*left*) and the histogram showing the wound healing rate (*right*). *E*, colony formation assay demonstrating the changes of proliferative potential in KYSE30 cells following SIRT3 KO. The colonies were quantified with ImageJ software and shown in mean ± SEM (n = 8). *F* and *G*, transwell assay analysis displaying an alternative migrated and invasion capacity of SIRT3 KO in KYSE30 cells (*F*) and the histogram showing their migrated efficiencies (*G*). *H*–*K*, SIRT3 KO and control (vector) KYSE30 cells were injected subcutaneously in BALB/C nude mice (*H* and *I*). Tumors were dissected and photographed after 23 days (*J*). Tumors were measured and graphed (*K*). ∗*p* < 0.05, ∗∗*p* < 0.01, ∗∗∗*p* < 0.001, ∗∗∗∗*p* < 0.0001. Data are mean ± SEM. Data are representative of three biological replicates. CCK-8, Cell Counting Kit-8; ESCC, esophageal squamous cancer cells; TCGA, The Cancer Genome Atlas.

SIRT3 is associated with a variety of cancers (41, 42, 43, 44), and in many cancers, the loss of SIRT3 triggers metabolic reprogramming that promotes tumorigenesis (27, 45, 46, 47, 48). The RNA-seq analysis revealed that SIRT3 is lowlier expressed in ESCC tumor tissues than that in normal tissues (Fig. 2*A*), indicating that SIRT3 expression is negatively correlated with the development of ESCC. The decreased expression of SIRT3 in EC suggests that SIRT3 has a tumor suppressive effect in ESCC. We also used the RNA-seq data from The Cancer Genome Atlas database and identified four SIRT3-associated pathways that were significantly downregulated in EC tissue. We speculate that these four SIRT3-related pathways are directly related to the occurrence and development of EC (Fig. 2*B*). To explore the role of SIRT3 during ESCC tumorigenesis, we first performed a series of malignant phenotype-associated assays. Increasing efficiencies of proliferation and wound healing rate were observed in all SIRT3 KO ESCC cells (Fig. 2, *C* and *D*). Additionally, low expression of SIRT3 in KYSE30 largely increased colony formation (Fig. 2*E*) and demonstrated a stronger migrated and invasion ability (Fig. 2, *F* and *G*). Collectively, SIRT3 is a strong tumor suppressor molecule during malignant progression in ESCC cells. To evaluate the impact of SIRT3 on tumor-forming capacity *in vivo*, we transplanted subcutaneously SIRT3-KO and control KYSE30 cells into nude mice, then we found that decreasing SIRT3 greatly contributed to a stronger tumorigenic potential than control group as reflected by increased frequency of tumorigenic cells (Fig. 2, *H*–*K*).

### SIRT3 Recognizes and Binds Histone H3K9la Mark

**Fig. 3:**
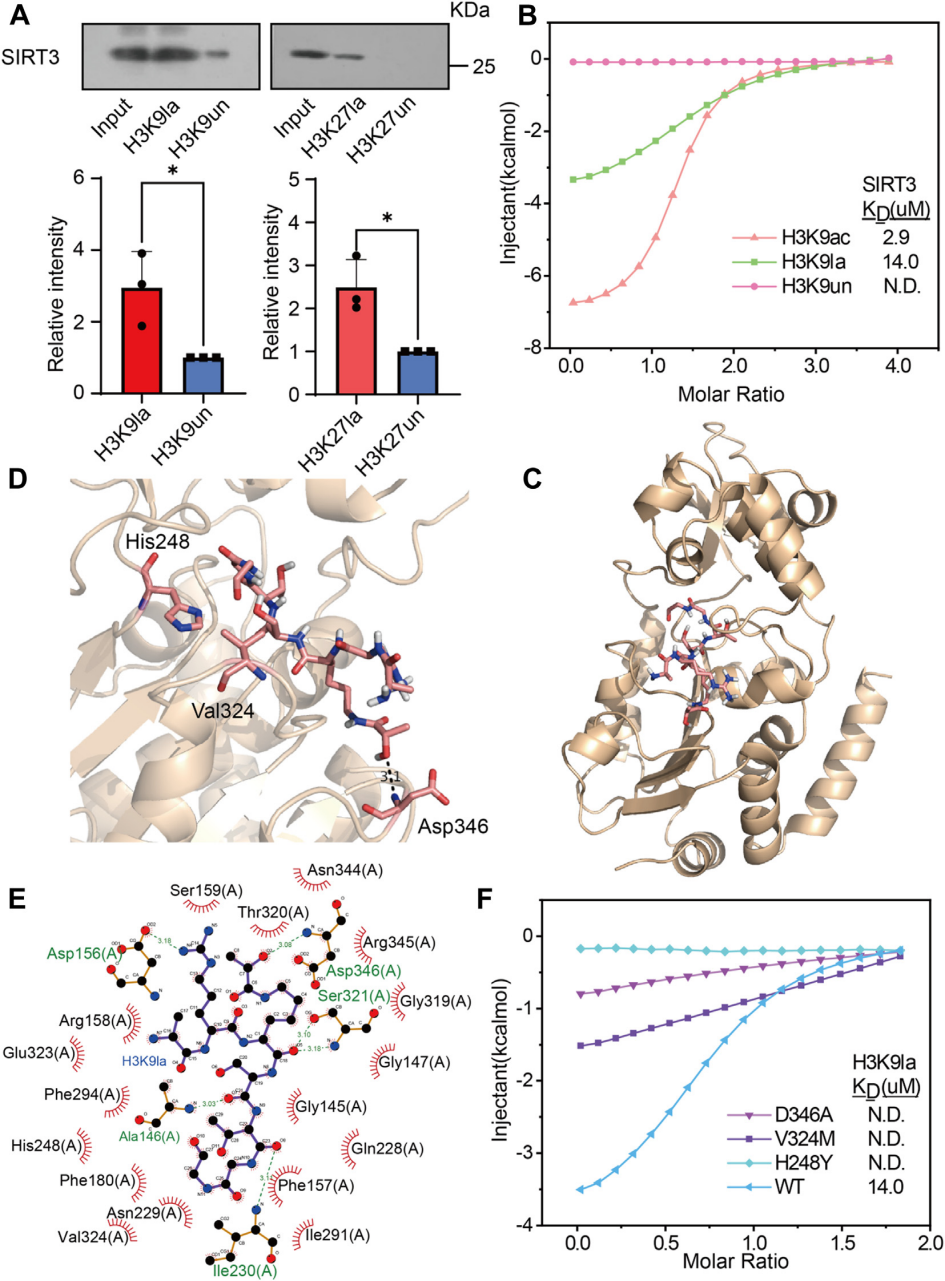
**Identification of SIRT3 as a selective and tight binding partner of lysine-9 lactylated histone H3**. *A*, recombinant SIRT3 was selectively pulled down *in vitro* by peptide H3K9la and showed weak binding activity toward peptide H3K27la. Relative intensity of SIRT3 in two groups were generated by the ImageJ software, respectively (n = 3 each). ∗*p* < 0.05, Student's *t* test. *B*, isothermal titration calorimetry measurement for the binding affinity of SIRT3 toward the H3K9ac, H3K9la, and H3K9un peptide. *C*, overall molecular docking structure of the complex of SIRT3 (*golden*) with H3K9la peptide (*gray*). *D*, molecular docking structure of the complex of SIRT3 (*golden*) with H3K9la peptide (*gray*) in surface form. *E*, LIGPLOT diagram listing critical contacts between the H3K9la peptide and SIRT3. H3 segment (*purple*) and key residues of SIRT3 (*brown*) are depicted in ball-and-stick mode. *Black ball*, carbon; *blue ball*, nitrogen; *red ball*, oxygen; *magenta dash line*, direct interaction hydrogen bond. *F*, isothermal titration calorimetry measurement for the binding affinity of SIRT3 and its catalytic mutants toward the H3K9la peptide.

Given that SIRT3 has the strongest affinity to H3K9bhb among human sirtuins (18), and the similarity of la and bhb mark in chemical structure (supplemental Fig. S3*B*), we reasonably speculated that SIRT3 also has a strong affinity for H3K9la. We thus selected SIRT3 for in-depth enzyme and structural characterization. We expressed and purified the NAD^+^ dependent deacetylase domain (18–399 aa) of human SIRT3 and investigated *in vitro* whether SIRT3 can directly and selectively bind to this lactylation histone peptide. As shown in Figure 3*A*, after the peptide pull down, SIRT3 was found to bind strongly to the H3K9la peptide, but weakly to the H3K27la peptide (Fig. 3*A* and supplemental Fig. S3*A*), confirming the direct and selective interaction between the recombinant protein SIRT3 and the H3K9la peptide. Furthermore, the binding affinity was directly measured in the absence of NAD^+^ using ITC, showing that SIRT3 binds to the H3K9la peptide with Kd = 14.0 μM, but not to the H3K9un peptide, and the binding of SIRT3 to H3K9ac acts as a positive control (Fig. 3*B*). To decipher the structural basis of SIRT3 recognition and binding to H3K9la, we used molecular docking to simulate the structure of H3K9la (7–12aa) peptide-bound SIRT3 in a binary complex lacking NAD^+^ state (Fig. 3*C* and supplemental Fig. S3*C*). SIRT3 adopts the classic sirtuin fold, consisting of lobular and lobular lobes arranged into Rossmann folds (49). The histone peptide binds in cracks formed at the interface of the two leaves. Consistent with previous reports, most of the peptide binding surfaces are negatively charged, which facilitates electrostatic binding of basic histone peptides (supplemental Fig. S3*D*). The H3K9la peptide is tightly attached to the substrate binding pocket, and the H3 peptide has additional hydrophobicity and hydrogen bond formation capabilities. H3K9la interacts with SIRT3 residues F294, V324, F180, T320, and H248 through hydrophobic interactions, and with residues D346, D156, and S321 through hydrogen bonds (Fig. 3*D*), and these hydrophobic interactions and hydrogen bonds contribute to specific recognition patterns. The LIGPLOT (50) lists the key contacts between the H3K9la peptide and SIRT3 (Fig. 3*E*). In addition, other H3 side-chain mediated hydrogen bonds involving R8_(NH)_:D156_(CO)_, S10_(CO)_:A146_(NH)_, and T11_(CO)_:I230_(NH)_ further strengthened the additional binding capacity. To verify the functional importance of pocket residues, corresponding SIRT3 mutants were generated and their binding activity to H3K9la peptide was measured by ITC. As shown in Figure 3*F*, the mutation of the catalytic residue H248Y almost completely eliminates its activity, demonstrating its important role in substrate recognition. D346A and V324M also adversely affect the binding of SIRT3 to H3K9la. These results demonstrate that the residues play an important role in the recognition and hydrolysis of Kla by SIRT3. The results of SIRT3 by recognizing the Kla site and its surrounding residues and catalyzing the lactylation peptide were also supported by extensive hydrophobic and hydrogen bonding interactions between SIRT3 and peptide side chains in the H3K9la complex.

### SIRT3 as an H3K9la Eraser to Regulate Gene Expression on Defined Chromatin Regions

**Fig. 4:**
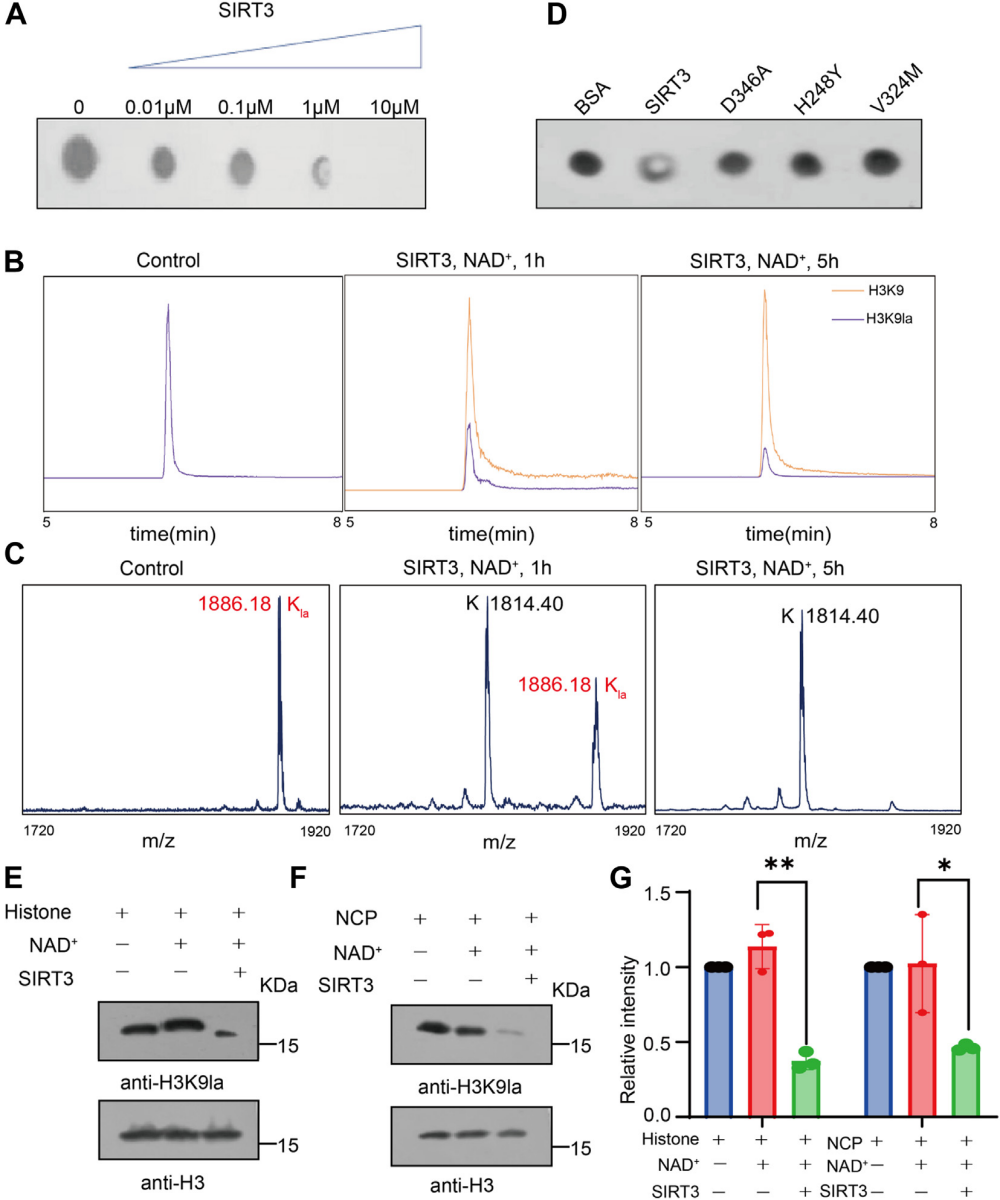
**SIRT3 catalyzes the hydrolysis of lactyl-lysine -lysine *in vitro***. *A*, Dot-blot based delactylation assays of SIRT3 with H3K9la peptide as substrate. *B* and *C*, the hydrolysis of the lactylated peptides by SIRT3 was analyzed by liquid chromatography–mass spectrometry *B*, MALDI-TOF MS based delactylation assays of SIRT3 with H3K9la peptide as substrate (*C*). The hydrolysis of H3K9la was observed with SIRT3 in the presence (*middle* and *right*), but not absence of nicotinamide adenine dinucleotide (NAD+) (*left*). *Orange* traces show ion intensity for the masses of delactylated (unmodified) peptides; and *violet* traces show ion intensity for the masses of lactylated peptides. *D*, Dot blot-based delactylation assays of WT and mutants SIRT3 with H3K9la peptide as substrate. *E* and *F*, immunoblotting results of deacylation assays on Kla-modified histones and nucleosome by with or without cofactor NAD^+^ and SIRT3. NCP, nucleosome core particle. *G*, relative level of H3K9 lactylation in three groups were generated by the ImageJ software, respectively (n = 3 each). ∗*p* < 0.05, ∗∗*p* < 0.01, Student's *t* test.

Inspired by SIRT3 binding lactyl-lysine in its catalytic pocket, we next tested whether SIRT3 has delactylation activity. Then, dot blotting, MALDI-TOF mass spectrometry and liquid chromatography-mass spectrometry (LC-MS) were used to detect the delactylation activity of SIRT3 to H3K9la (Fig. 4, *A*–*C*). As expected, SIRT3 removed lactyl groups from H3K9la in a time-, enzyme dose-, and NAD^+^ cofactor-dependent manner. For the residue H248 (H248F), which is essential for the catalytic deacetylation of SIRT3, the mutation completely eliminated its delactylation activity (Fig. 4*D*), suggesting that the mechanism by which SIRT3 catalyzes the hydrolysis of Kla is similar to that of Kac. To test the delactylation activity of SIRT3 on histones and nucleosomes, we extracted histones and nucleosomes from HEK 293T cells adding NaLa (supplemental Fig. S4, *B*–*D*) and incubated them with SIRT3in the presence of NAD^+^. Western blot was used with anti-H3K9la antibody to assess the lactylation level. Significantly reduced Kla levels were observed in bands with a molecular mass of about 15 kDa (Fig. 4, *E*–*G* and supplemental Fig. S4*A*). The results showed that SIRT3 can catalyze the removal of Kla of histone peptide, core histone, and nucleosomes. In general, the binding capacity can be well converted into delactylation enzyme activity. Together, our structural analysis, binding measurement, and enzymatic activity studies demonstrate the molecular basis for the delactylation of histone H3K9la by SIRT3 *in vitro*.

**Fig. 5:**
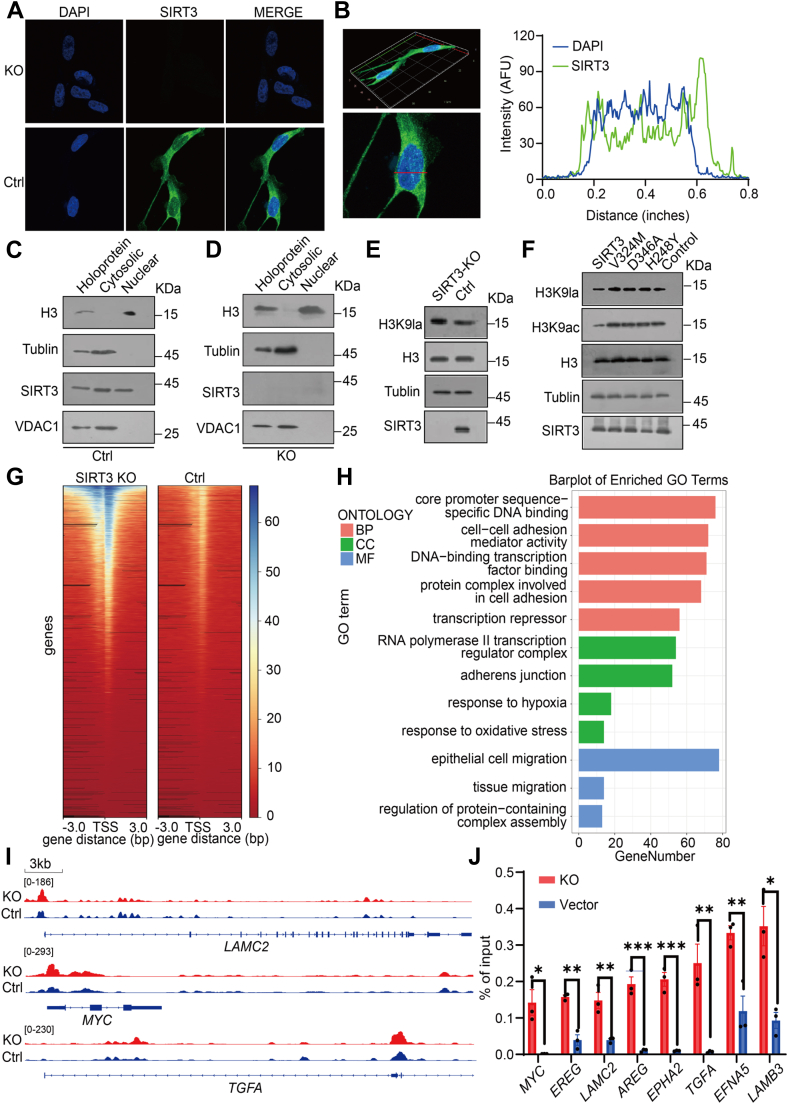
**SIRT3 regulates H3K9la levels and gene expression on its defined chromatin regions**. *A*, fluorescence analysis of SIRT3 in KYSE30 cells. *B*, KYSE cells were analyzed with DAPI (*blue*) and SIRT3 (*green*) and scanned along the *z*-axis using confocal microscopy (*top* image). The fluorescence intensities mid-lane in the cell in the *XY* axis are shown (*bottom* and *right* images). *C*, Western blots of KYSE30 cell extracted fractions. *D*, Western blots of KYSE30 cell extracted fractions after SIRT3 KO. *E*, Western blots of increasing H3K9 lactylation level after SIRT3 KO in KYSE30 cells. *F*, Western blot analysis showing that SIRT3 catalytic mutants overexpressed in KYSE30 cells caused the accumulations of H3K9la and H3K9ac levels. Relative level of H3K9la and H3K9ac in five groups were generated by the ImageJ software, respectively. Data are mean ± SEM (n = 3 each). ∗∗∗∗*p* < 0.0001, Student's *t* test. *G*, the binding density of H3K9la visualized by deepTools: Heat maps show differential binding peaks of H3K9la distributed within ±3kb in the TSS region in SIRT3 knock out cells and HEK 293T cells. *H*, functional enrichment analysis comparing the gene expression between control and SIRT3 KO cells. The differentially expressed genes were enriched in GO database and visualized through bar plot using the R package clusterProfiler. *I*, representative traces of ChIP-seq showed that H3K9la was enriched in the TSS region of *LAMC2*, *MYC*, and *TGFA* genes. *Red tracks* represent the peak of SIRT3 KO cell enrichment, while *blue tracks* represent the peak of ESCC KYSE30 cell enrichment. *J*, ChIP-qPCR results for *LAMC2*, *MYC*, and *TGFA* genes. The bar graph results showed promoter levels enriched with H3K9la in control and SIRT3 KO cells. Data are mean ± SEM (n = 3). ∗*p* < 0.05, ∗∗*p* < 0.01, ∗∗∗*p* < 0.001. ChIP-qPCR, chromatin precipitation quantitative PCR; DAPI, 4',6-diamidino-2-phenylindole; ESCC, esophageal squamous cancer cells; GO, gene ontology; TSS, transcription start site.

**Fig. 6:**
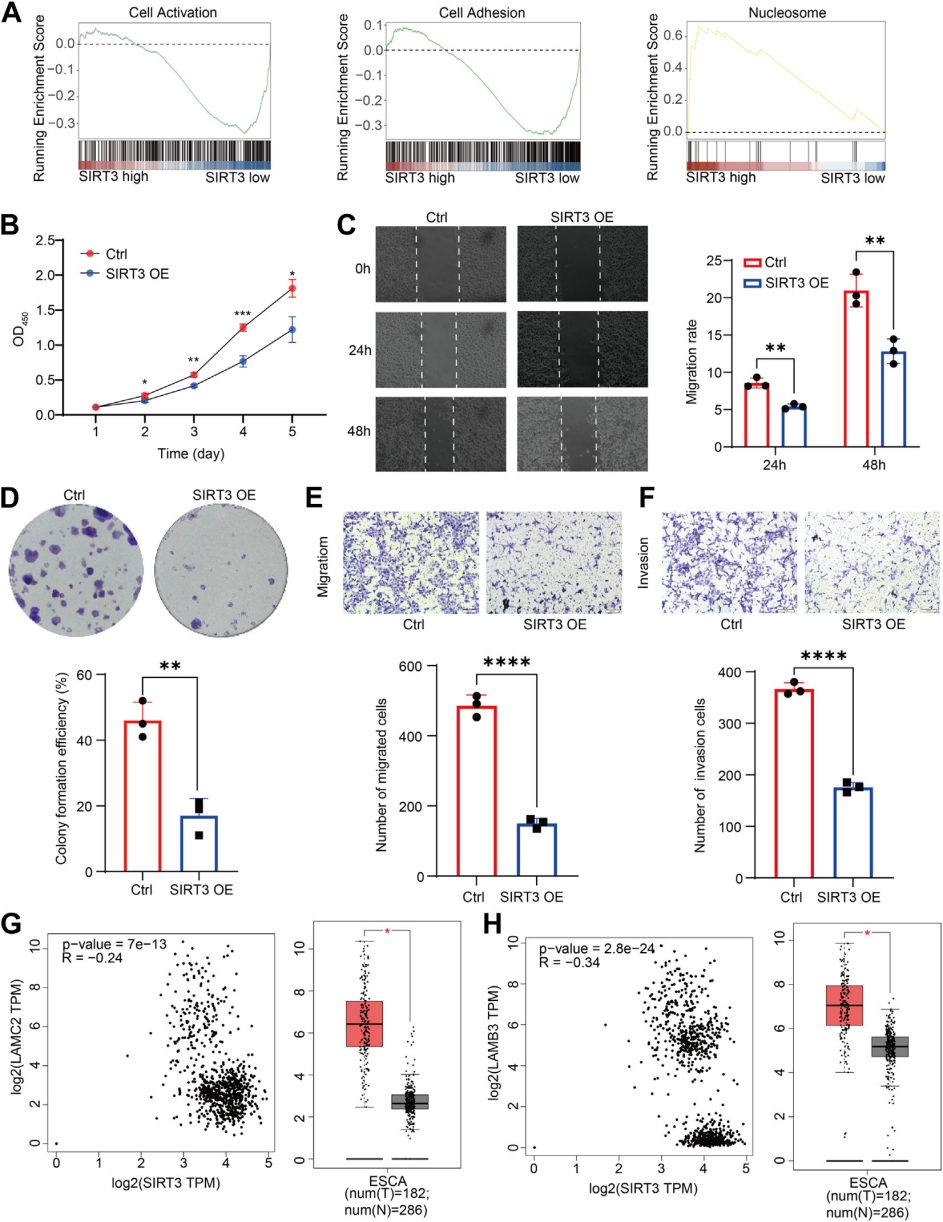
**SIRT3 inhibits proliferation and migration of ESCC cells by regulating H3K9la**. *A*, gene Set Enrichment Analysis (GSEA) for regulation of cell activation, cell adhesion, nucleosome pathways in cancer were analyzed from ChIP-seq data. *B*, CCK-8 analysis of the viability of KYSE30 cells when overexpression SIRT3. The quantification of the *A*_450_ were shown in mean ± SEM (n = 8); ∗*p* < 0.05. Data are representative of three biological replicates. *C*, scratch wound healing assay results suggesting the impact of SIRT3 overexpression on the wound healing ability in KYSE30 cells (*left*) and the histogram showing the wound healing rate (*right*). *D*, colony formation assay demonstrating the changes of proliferative potential in KYSE30 cells following SIRT3 overexpression. The colonies were quantified with ImageJ software and shown in mean ± SEM (n = 8). *E* and *F*, transwell assay analysis displaying an alternative migrated and invasion capacity of SIRT3 overexpression in KYSE30 cells (*E*) and the histogram showing their migrated efficiencies (*F*). *G* and *H*, transcriptional analysis of tissue samples from ESCC patients using GSEA database. CCK-8, Cell Counting Kit-8; ChIP-seq, chromatin immunoprecipitation sequencing; ESCC, esophageal squamous cancer cells; GSEA, Gene Set Enrichment Analysis.

We wonder if SIRT3 can enter into nucleus and specifically modulate H3K9la for the gene transcription. Confocal images showed that the fluorescence signal of SIRT3 was clear in the nucleus but brighter in the mitochondria (Fig. 5, *A* and *B*). Cytoplasmic separation experiments showed that SIRT3 could be detected in both cytoplasm and nucleus, while no detection after SIRT3 KO (Fig. 5, *C* and *D* and supplemental Fig. S2, *A* and *B*), and mitochondrial voltage-dependent anion channel 1 (VDAC1) protein excluded the mitochondrial contamination in nuclear fraction, indicating that SIRT3 can directly localize on chromatin. Next, we investigated whether SIRT3 regulates histone Kla in cells. 3-TYP is a selective SIRT3 inhibitor (51) (supplemental Fig. S2*E*). We stimulated cells with the inhibitor 3-TYP, and compared with dimethyl sulfoxide, the level of H3K9la in the 3-TYP group was significantly increased (supplemental Fig. S2*F*). Increasing efficiencies of proliferation and colony formation were also observed with inhibitor 3-TYP in ESCC cell. This suggests that SIRT3 inhibits ESCC cell proliferation and migration through its delactylation activity (supplemental Fig. S2, *G* and *H*). To analyze the intracellular delactylation activity of SIRT3, we knocked down SIRT3, and the level of H3K9la was significantly increased (Fig. 5*E* and supplemental Fig. S2*C*). Furthermore SIRT3 WT and each mutant plasmids were transfected, and the results showed that WT SIRT3 had higher delactylation activity than catalytic mutants (Fig. 5*F* and supplemental Fig. S2*D*). We hypothesized that SIRT3 could regulate gene transcription by controlling histone H3K9la levels. To test this hypothesis, we performed ChIP-seq and observed that H3K9la modulated by SIRT3 is highly enriched in gene transcription start sites (TSSs) (Fig. 5*G* and supplemental Fig. S5*A*), suggesting that SIRT3 plays a role in transcriptional inhibition. A total of 6065 genes were upregulated after SIRT3 KO. GO analysis and KEGG analysis further indicated that these upregulated genes were mainly related to tumorigenesis and development (Fig. 5*H* and supplemental Fig. S5*B*), which was consistent with the increase of cell proliferation after SIRT3 inhibition. We focused on eight candidate genes including *LAMC2* (52, 53), *MYC* (54, 55, 56), *TGFA* (57, 58), and *LAMB3* (59, 60) whose TSSs were close to H3K9la rich regions (Fig. 5*I* and supplemental Fig. S5*D*). A combination of ChIP-qPCR (Fig. 5*J*) was used to detect Kla levels near the TSS of candidate genes. As shown in Figure 5*J*, knocking out of SIRT3 resulted in significantly increasing Kla levels in the eight genes analyzed, suggesting that SIRT3 may directly regulate lactylation kinetics at genomic sites to which it binds. Given that histone Kla is enriched at active gene promoters and potential enhancers (61), the negative correlation between gene transcription levels and nearby histone Kla levels at SIRT3 KO suggests that SIRT3 may exert inhibitory effects on these target genes by "erasing" histone Kla marks. Gene Set Enrichment Analysis from ChIP-seq data (Fig. 6*A* and supplemental Fig. S5*C*) indicated SIRT3 regulated cell activation, cell adhesion, and nucleosome pathways in cancer through H3K9la levels.

### SIRT3 Inhibits Proliferation and Migration of ESCC Cells by Increasing the Transcription of Tumor Suppressor Genes Regulated by the H3K9la

Given that investigations into the effects of SIRT3-reguated histone Kla on tumors are limited, SIRT3's role in regulating tumor initiation and progression remains unclear. To further examine whether the delactylation activity of SIRT3 to H3K9la contributes to the inhibition of ESCC cell growth, we overexpressed SIRT3 in KYSE30 cells and performed Cell Counting Kit-8 assay and colony formation assay. Clearly, increased SIRT3 led to a reduced proliferation and colony-forming rate (Fig. 6, *B* and *C*). As expected, decreased wound healing efficiency (Fig. 6*D*) and lower migrated and invasion rates (Fig. 6, *E* and *F*) were observed in the cells with overexpressed SIRT3. Transcriptional analysis of tissue samples from ESCC patients using Gene Set Enrichment Analysis database showed that oncogenes regulated by SIRT3 were highly expressed in ESCC tissues and were negatively correlated with the expression level of SIRT3 (Fig 6, *G* and *H* and supplemental Fig. S6, *A*–*F*). Therefore, low SIRT3 expression enhances tumorigenicity of ESCC cells. These results indicated that the Kla on H3K9 is essential for its role in promoting ESCC cell growth and SIRT3 could suppress ESCC cell growth by regulating H3K9la level. Overall, our results provide an evidence that SIRT3 acts as a tumor suppressor factor to inhibit the transcription of genes associated with tumorigenesis and development by reducing histone lactylation levels. Whether the characterization of H3K9la and SIRT3 levels can be used as biomarkers to predict the clinical outcome and therapeutic targets of EC will be a very promising research direction.

## Discussion

With the rapid development of the detection technology, more and more novel histone lysine acylation forms have been discovered (62), and thereby their regulatory factors and functional consequence have received widespread attention. However, there is still a lack of reliable methods to unbiasedly identify the interactors (such as readers, enzymes, and cofactors) of the histone PTMs *in vivo,* because the interaction between PTMs and its regulatory factors is weak and transient, limiting the applicability of traditional biochemical pull-down methods (63). Based on this, we applied a chemical proteomics approach based on antibody-mediated proximity labeling for profiling the interactome of histone H3K9la. By intracellular interactive networks fixing, antibody-mediated localization, and biotinylation, the landscape of H3K9la interactors was revealed. This approach not only allows to find PTM "readers" involved in relatively more stable protein-protein interactions (64), but also to decipher weak and transient enzyme-PTM interactions. Indeed, we identified the erasers and cofactors of histone Kla, such as HDAC1 with MAT1 and MAT2, HDAC2 with SIN3. Notably, we found that SIRT3 is highly preferred to H3K9la, and has delactylation activity by structural analysis, binding force measurement, and enzymatic activity study, which further were verified from three level experiments including peptide, histone protein, and nucleosome.

There is increasing evidence that short chain acylation of histone lysine contributes to the regulation of chromatin structure and gene expression (65, 66). With a hydroxyl group, lysine lactylation is similar to β-hydroxybutyrylation and 2-hydroxyisobutyrylation in chemical property. However, they are regulated by distinct metabolic pathways and associated with unique physiology and disease (67, 68). In eukaryotes, histone acylation is an important epigenetic mark that regulates gene expression by addition or removal of acylation enzyme-catalyzed, loosing or tightening the interactions between DNA and histones. Sirtuins are NAD^+^-dependent deacetylases, and emerging evidence suggests that acylation on histone lysine residues are dynamically regulated under various physiological and pathological conditions. In this study, we demonstrated that human SIRT3 catalyzes the hydrolysis of histone lactylation. By molecular docking, we found that SIRT3 has a hydrophobic pocket lined with hydrogen bonds for the recognition and catalysis of Kla, while lactyl-group can form hydrogen bond with D346 of SIRT3, which has unique stability. In addition, T320, H248, and V324 contribute to critical hydrophobic contact with histone H3. It provides a new understanding of H3K9la-mediated cellular function and mechanism regulated by SIRT3.

SIRT3 is a key regulator of the Warburg effect in mitochondria. In various types of cancers, the loss of SIRT3 triggers the reprogramming of glycolytic metabolism and increases the level of ATP and the content of lactate, thus promoting tumorigenesis (69, 70). In this study, we demonstrated that endogenous SIRT3 acts as an "eraser" to regulate histone lactylation in nucleus, and knocking out SIRT3 or using inhibitor 3-TYP in cells confirms these results. This discovery opens up a new opportunity to study the cellular functions of protein lactylation. Mounting evidence suggests that various histone acylation are mainly associated with active gene transcription (71, 72). Based on the analysis of multiple SIRT3 target gene loci, this study further indicates that there is a potential correlation between SIRT3 KO and transcriptional upregulation and local histone Kla level increasing. Thus, it gives rise to the hypothesis that SIRT3 might cause silencing by "erasing" Kla on target genes. To test this hypothesis and examine the correlation between SIRT3-catalyzed histone delactylation and gene expression, we performed ChIP-seq on control and SIRT3 KO cells, and conducted a comprehensive genome-wide analysis of gene expression regulated by histone H3K9la and SIRT3. By analyzing the data set, we searched for genes that were significantly upregulated in SIRT3 KO cells, such as *MYC*, *LAMC2*, *TGFA*, and *LAMB3*. It is important for us to study the effect of site-specific histone Kla marks targeted by SIRT3 on gene expression regulation. Together, these findings not only provide a new insight into the novel activity of SIRT3, but also validate the regulation of histone Kla by a pharmacologically targetable enzyme, laying the foundation for future functional studies of this modification.

SIRT3 is closely associated with many different kinds of solid tumors (41, 42, 43, 44), given that histone lactylation can activate gene transcription and drive tumorigenesis; we hypothesized that knocking out SIRT3 or reduced SIRT3 activity may lead to increased histone lactylation in cells, thereby affecting tumor progression. SIRT3 has been reported as a tumor suppressor in studies of various human cancers by triggering metabolic reprogramming that promotes tumorigenesis (27, 45, 46, 47, 48), of which EC is of particular interest to clinicians because it is the most common solid tumor and the leading cause of cancer death. Specific SIRT3 inhibitor (3-TYP) leads to the increased H3K9la levels. Similarly, SIRT3-catalyzed mutant H248Y significantly hindered the delactylation activity of SIRT3, further confirming that SIRT3 is a histone delactylation enzyme. Functional analysis showed that the cell proliferation rate and cell migration ability were significantly increased after the inhibition of SIRT3 by inhibitor 3-TYP, suggesting that the increase in cell proliferation rate caused by SIRT3 inhibition depended on intracellular H3K9la level.

In this study, we identified the interactome of H3K9la by an antibody-mediated proximity labeling approach and found that SIRT3 is a mayor histone delactylase by *in vitro* and *in vivo*. We further revealed the regulatory mechanism underlying H3K9la regulated by SIRT3 and its potential biological consequences on gene transcription. In addition, the potential function of abnormal histone lactylation on the progression of KYSE30 was predicted. SIRT3 can restrain the product of lactate in mitochondria to decrease histone lactylation indirectly, more importantly, remove histone lactylation directly in nucleus. It is interesting that SIRT3 plays dual effects on suppression of lysine lactylation in mitochondria and nucleus. Therefore, inhibiting SIRT3 delactylation function may provide a unique opportunity for innovative antitumor therapies targeting ESCC cells.

## Data Availability

The mass spectrometry proteomics data have been deposited to the ProteomeXchange Consortium with the dataset identifier PXD061251. ChIP-seq data are available through GEO (GSE274648).

## Supplementary data

Supplementary Data are available at Molecular & cellular proteomics online. This article contains supplemental data.

## Conflict of interest

The authors declare no competing interests.
