# Supplementary material for: SIRT3 Functions as an Eraser of Histone H3K9 Lactylation to Modulate Transcription for Inhibiting the Progression of Esophageal Cancer
Source: Mol Cell Proteomics. 2025 Apr 17;24(5):100973. doi: 10.1016/j.mcpro.2025.100973 (PMC12144510; doi:10.1016/j.mcpro.2025.100973)
Supplement: Supporting information [file mmc1.pdf]

Supporting Information for

SIRT3 functions as an eraser of histone H3K9 lactylation to modulate transcription for inhibiting the progression of esophageal cancer

Chen Chen[1,*,†], Yingao Zhang[1,†], Yong Zang[1,2†], Zilong Fan[1,3], Yanpu Han[1], Xue Bai[1], Aiyuan Wang[1,3], Jianji Zhang[1], Ju Wang[2], Kai Zhang[1,3,4*]

[1] Key Laboratory of Breast Cancer Prevention and Therapy (Ministry of Education), Key Laboratory of Immune Microenvironment and Disease (Ministry of Education), The Province and Ministry Co-sponsored Collaborative Innovation Center for Medical Epigenetics, School of Basic Medical Sciences, Tianjin Medical University Cancer Institute and Hospital, Tianjin Medical University, Tianjin, China

[2] School of Biomedical Engineer, Tianjin Medical University, Tianjin, China

[3] Department of Bioinformatics, Tianjin Key Laboratory of Medical Epigenetics, Tianjin Medical University, Tianjin, China

[4] State Key Laboratory of Experimental Hematology, Tianjin Medical University, Tianjin, China

* To whom correspondence should be addressed. Tel: +86 022 83336833; Email: kzhang@tmu.edu.cn or chench@tmu.edu.cn

† The first, second, and third authors should be regarded as Joint First Authors.

A

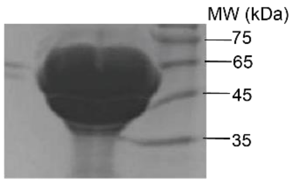

B

	Peptides	Coverage [%]	log_2FC	$-log_{10}p$-value
SIRT3	16	62	11.83	5.81
HDAC1	9	24	4.71	3.75

C

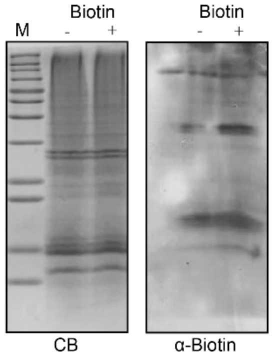

D

E

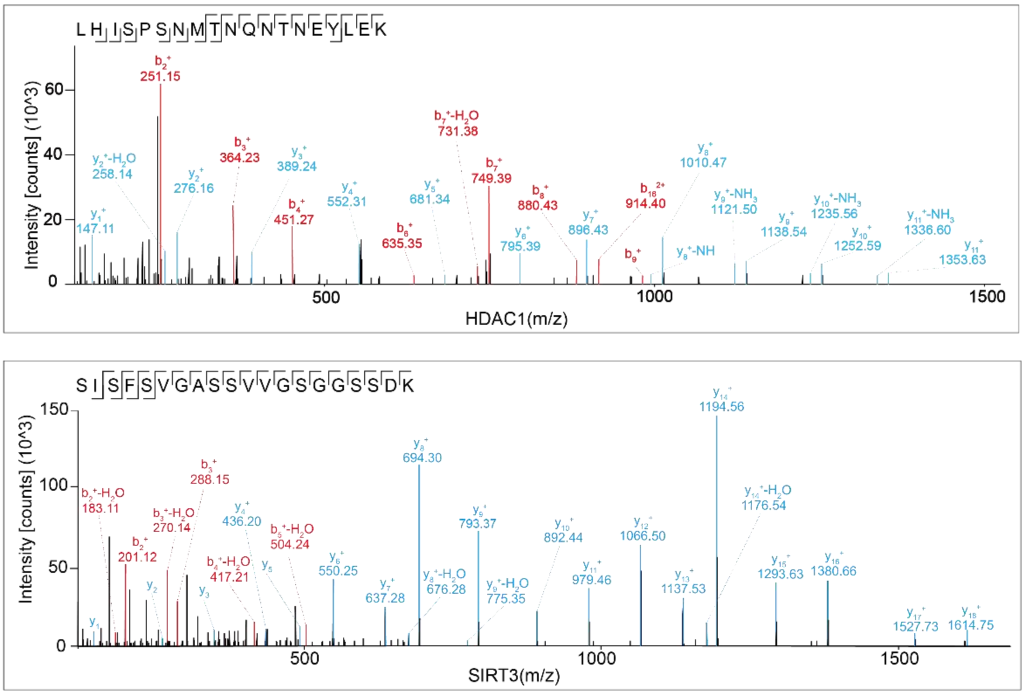

Figure S1. Antibody-mediated protein A-TurboID proximity labeling strategy. (A) SDS-PAGE of recombinant fusion protein TurboID-protein A expressed in *E.coli*. (B) Unique peptide numbers and coverage of candidate lactylation mark eraser proteins. (C) Immunoprecipitation of biotinylated proteins after targeting proteins. (D) Representative mass spectrometry (MS) spectra of peptide 343-LHISPSNMTNQNTNEYLEK-361 from HDAC1 identified in the experiment. (E) Representative mass spectrometry (MS) spectra of a peptide 101-SISFSVGASSVVGSGGSSDK-120 from SIRT3 identified in the experiment.

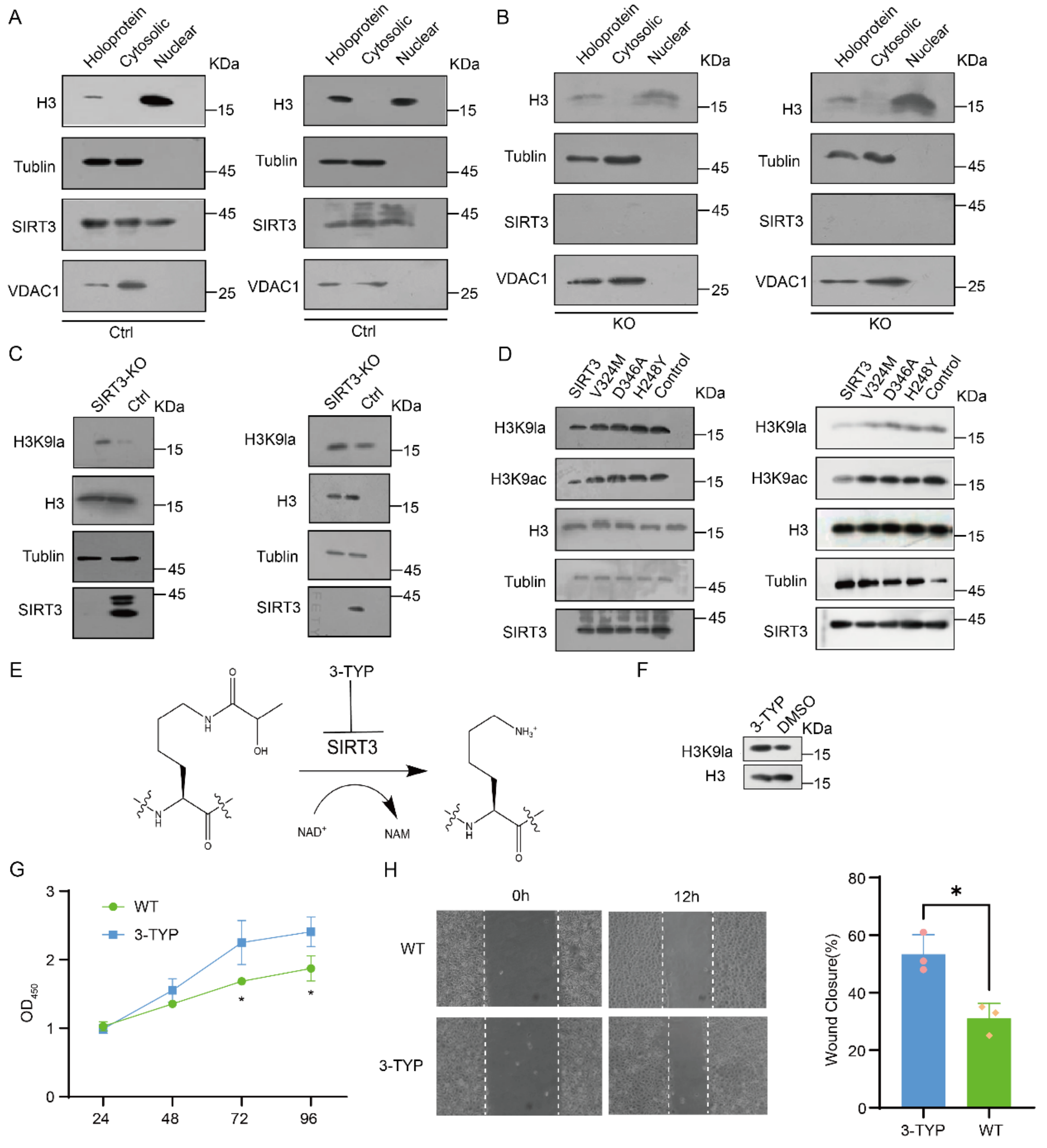

Figure S2. SIRT3 regulates H3K9la levels.

(A) Western blots of KYSE30 cell extracted fractions. Data are representative of three biological replicates. (B) Western blots of KYSE30 cell extracted fractions after SIRT3 KO. Data are representative of three biological replicates. (C) Western blots of increasing H3K9 lactylation level after SIRT3 KO in KYSE30 cells. (D) Western blot analysis showing that SIRT3 catalytic mutants overexpressed in KYSE30 cells caused the accumulations of H3K9la and

H3K9ac levels. Data are representative of three biological replicates. (E) Schematic diagram of the delactylation reaction catalyzed by SIRT3. (F) Western blot analysis showing the decreasing delactylation of SIRT3 with the specific inhibitor 3-TYP. (G) CCK8 analysis of the viability of KYSE30 cells when SIRT3 specific inhibitor 3-TYP was added. The quantification of the OD450 were shown in mean ± SEM (n = 8); *P < 0.05. Data are representative of three biological replicates. (H) The inhibitor 3×TYP treated cells and the non-treated cells were subjected to wound-healing assay. The images (left) and quantification (right) of the percentages of wound closure were shown in mean ± SEM (n = 8); *P < 0.05; Unpaired t-test. Data are representative of three biological replicates.

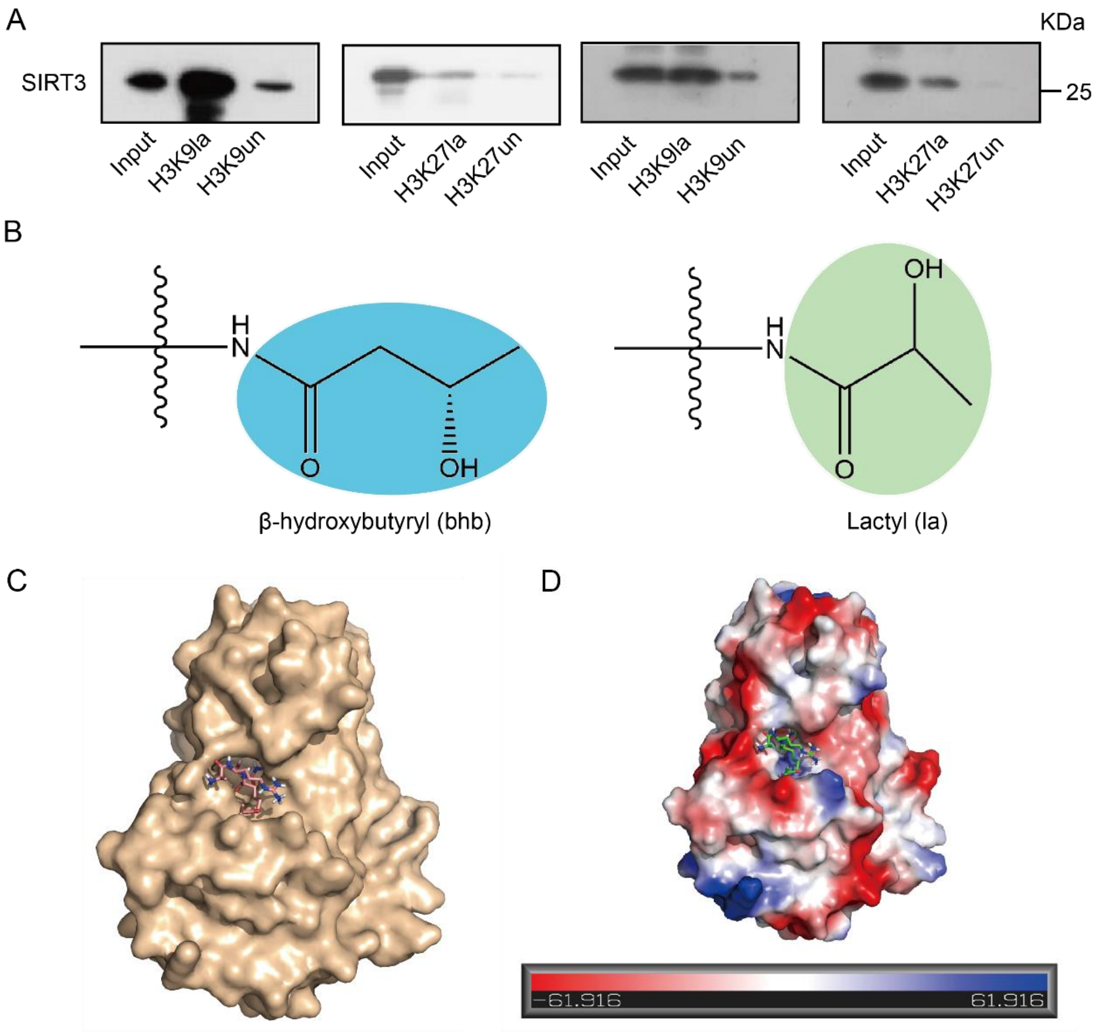

Figure S3. Identification of SIRT3 as a selective and tight binding partner of lysine-9 lactylated histone H3. (A) Recombinant SIRT3 was selectively pulled down in vitro by lactylated peptide H3K9la and H3K27la. Data are representative of three biological replicates. (B) Chemical structures of two similar acylations. Abbreviations of bhb and la acylation marks are used throughout this article. (C) Overall molecular docking structure of the complex of SIRT3 (golden) with H3K9la peptide (grey) in surface form. (D) Surface charge distribution analysis of SIRT3 protein in the peptide binding region.

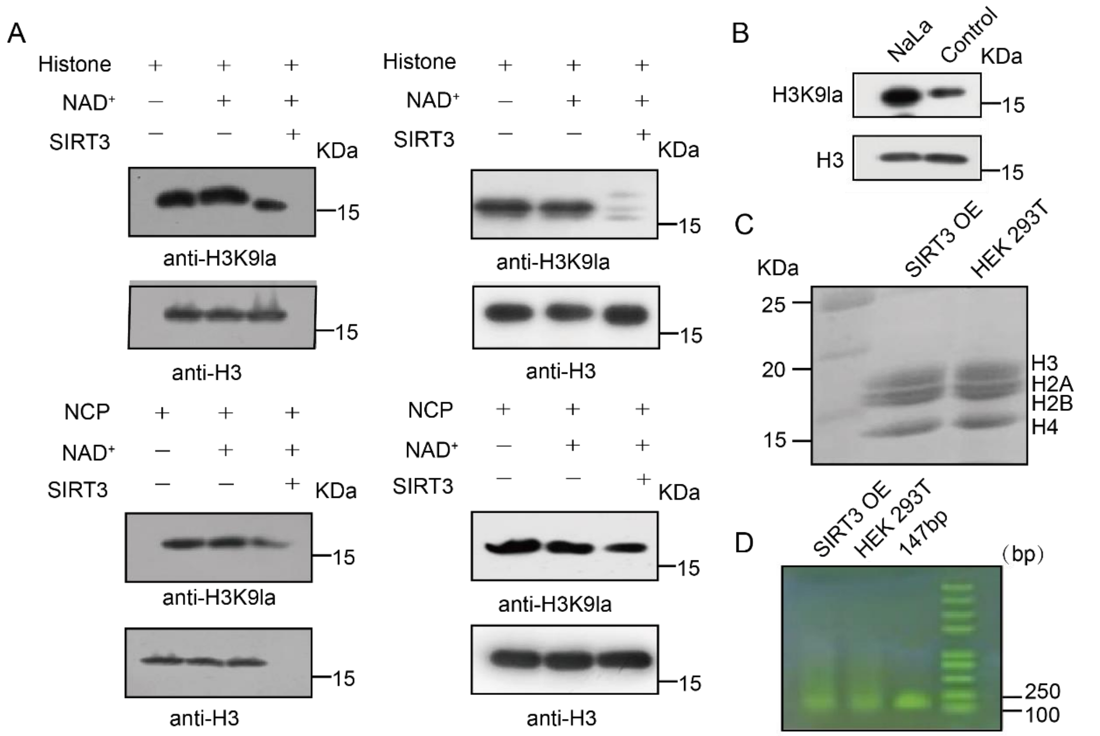

Figure S4. SIRT3 regulates histone lysine lactylation in vitro and in nucleus. (A) Immunoblotting results of deacylation assays on Kla-modified histones and nucleosome by with or without cofactor NAD+. Data are representative of three biological replicates. (B) After sodium lactate stimulation of HEK 293T cells, the H3K9la level increased. (C) Coomassie blue staining results of extracted nucleosomes from HEK 293T cells. NC, nucleosome. (D) Agarose gel eletrophoresis analyses showing that 147bp DNA twined nucleosomes extracted from HEK 293T cells. DNA length of 147bp was used as positive controls.

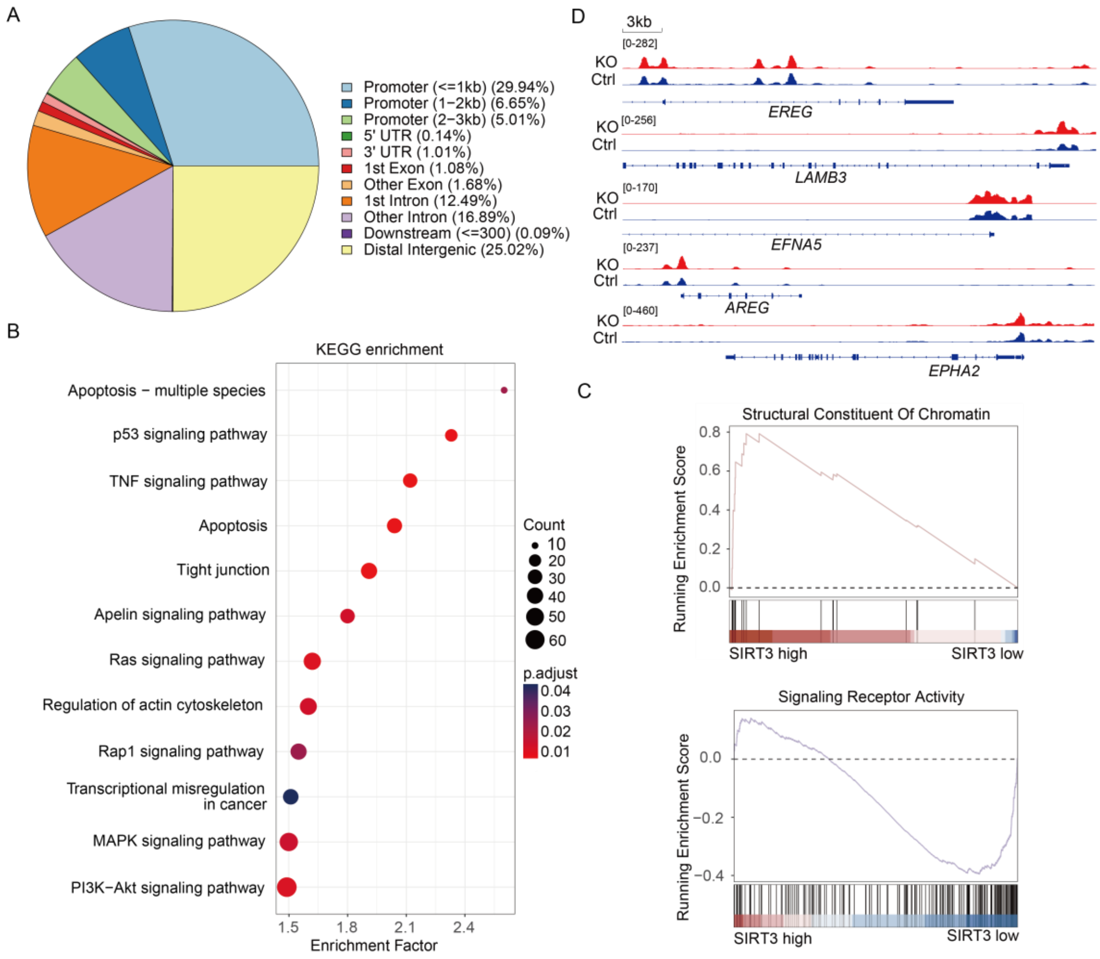

Figure S5. SIRT3 regulates gene expression regulated by H3K9la levels on its defined chromatin regions. (A) Genome-wide distribution of downregulated H3K9la-binding peaks in SIRT3 knockout KYSE30 cells. (B) The differentially expressed genes were enriched in KEGG pathway database and visualized through dot plot using the R package clusterProfiler. (C) Gene Set Enrichment Analysis (GSEA) for regulation of cell activation, cell adhesion, oxidative phosphorylation, nucleosome pathways in cancer were analyzed from ChIP-seq data. (D) Representative traces of ChIP-seq showed that H3K9la was enriched in the TSS region of EREG, LAMB3, EFNA5 and AREG genes. Red

tracks represent the peak of SIRT3 KO cell enrichment, while blue tracks represent the peak of ESCC KYSE30 cell enrichment.

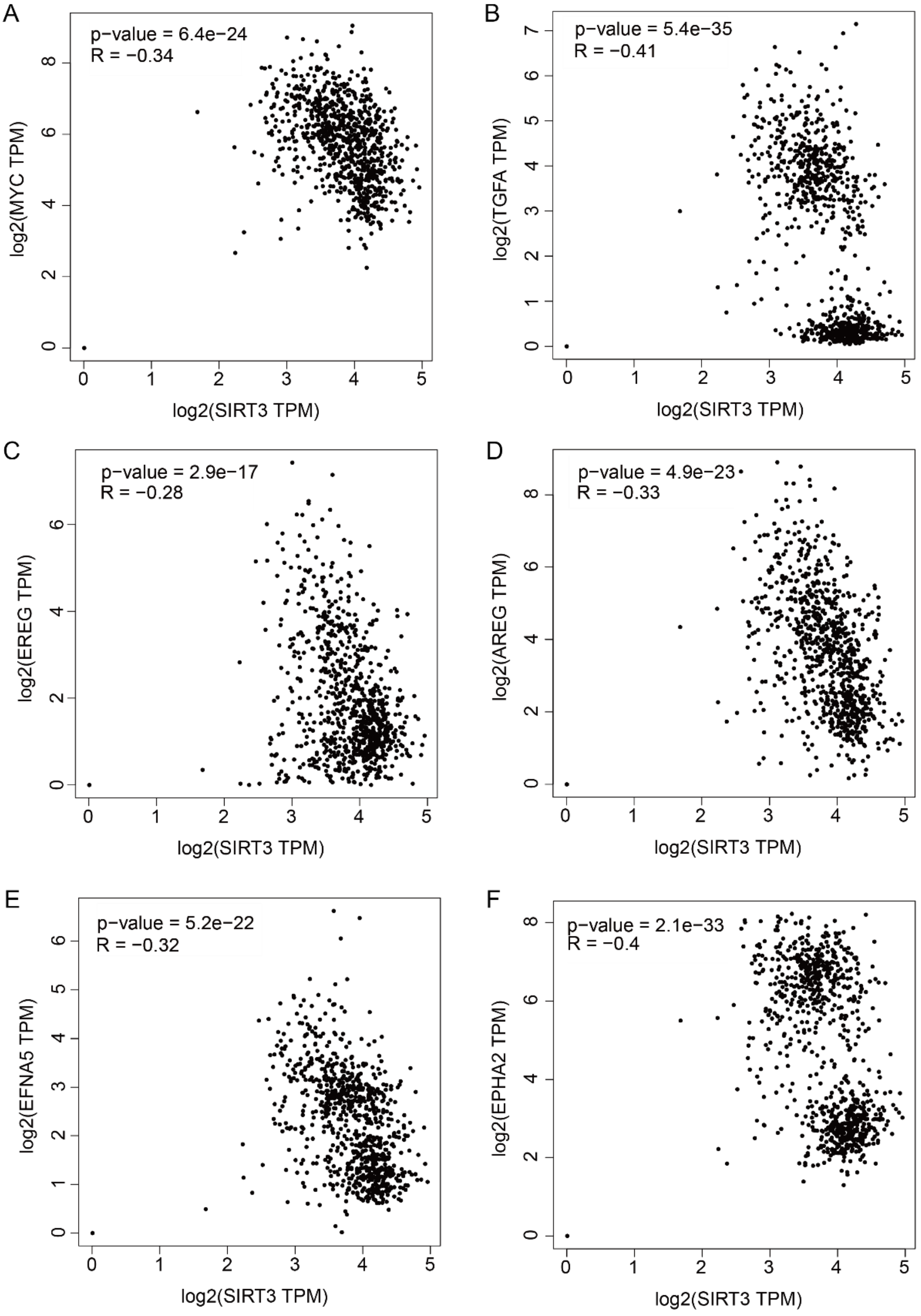

Figure S6. H3K9la related genes correlationship with SIRT3 in of ESCC tissues. (A-F) Correlation analysis of SIRT3 regulating genes and SIRT3 in esophageal cancer tissues derived from GEPIA database, *P < 0.05; Unpaired t-test.
